# Engineering biopolymer nanoparticles for targeted nanomedicine in cancer therapy and food preservation

**DOI:** 10.3389/fnut.2026.1778133

**Published:** 2026-04-30

**Authors:** Limin Zheng, Li-Choo Chong, Xiaosong Zhai

**Affiliations:** 1 Shandong Facility Horticulture Bioengineering Research Center, Jia Sixie College of Agriculture, Weifang University of Science and Technology, Weifang, China; 2 Taylor’s Culinary Institute, Faculty of Social Sciences and Leisure Management, Taylor’s University, Subang Jaya, Selangor, Malaysia

**Keywords:** therapy, carbohydrates, nanoparticles, food preservation, nanomedicine, nanotechnology

## Abstract

Cancer therapy and food preservation both face significant challenges related to stability, targeted delivery, safety, and efficiency of active compounds. Biopolymer-based nanoparticles, particularly those derived from chitosan and other carbohydrates, have emerged as versatile platforms capable of addressing these limitations in both biomedical and food systems. This review provides a comprehensive overview of the design, engineering, and surface functionalization of nanoparticles, highlighting their physicochemical properties, formulation strategies, and mechanisms governing stability, encapsulation efficiency, and controlled release. In the biomedical context, these nanocarriers have shown considerable promise for targeted cancer therapy by improving drug bioavailability, enhancing cellular uptake, enabling receptor-mediated targeting, and reducing systemic toxicity. Advances in ligand-mediated surface functionalization, active targeting strategies, and stimuli-responsive delivery systems are discussed, together with current progress in preclinical and clinical development of nanomedicines. At the same time, carbohydrate-based nanoparticles are increasingly explored in food science as delivery systems for bioactive compounds, antimicrobial agents, and natural preservatives, where they contribute to improved food stability, extended shelf life, and protection of sensitive nutrients. By integrating perspectives from nanomedicine and food technology, this review highlights the multifunctional potential of chitosan and carbohydrate-derived nanomaterials as sustainable and biocompatible platforms for controlled delivery in both therapeutic and food preservation applications. Critical challenges related to large-scale production, stability, regulatory considerations, and translational feasibility are also examined. Overall, advances in nanoparticle engineering and surface functionalization may enable the development of next-generation biopolymer nanocarriers with broad applications spanning targeted cancer therapy and innovative food preservation technologies.

## 1 Introduction

Cancer remains one of the leading causes of morbidity and mortality worldwide, with its global burden continuously increasing. Recent statistical analyses indicate that approximately 20 million new cancer cases and 9.7 million deaths were reported globally in 2022, and this number is projected to rise substantially in the coming decades, potentially exceeding 35 million cases by 2050 (1). Moreover, recent estimates for 2025 suggest that more than 2 million new cancer cases and over 600,000 deaths will occur in the United States alone, highlighting the ongoing escalation of cancer incidence (2). These statistics underscore the urgent need for more effective and targeted therapeutic strategies. Notwithstanding progress in cancer therapy, controlling the biological disposition of active pharmaceutical ingredients (APIs) after administration continues to pose a considerable challenge in the development of innovative therapeutics. Poor stability, solubility, and rapid metabolism, which lead to poor pharmacokinetics, are frequently the limitations of current first-line therapies, particularly in the field of cancer. Additionally, significant off-target effects cause harmful toxicities in healthy cells, which lowers treatment tolerability and worsens patient outcomes. Therefore, insufficient drug delivery to the tumor site, rather than the drug’s effectiveness, is often the cause of treatment failure (3). Adding the medication to Nano-based drug delivery systems is one way to get around this problem. Although it began with the idea of creating a better formulation for challenging or insoluble APIs, Nanomedicine is currently a rapidly developing multidisciplinary field. Numerous studies have been conducted to examine the design and development of nanoparticles, including evaluations of active targeting, triggered drug release, and passive targeting/accumulation (4). As a temporary barrier for labile therapeutics and generally poor solubility drugs, nanoparticles have one advantage in trapping APIs inside their structure, thus improving the bioavailability of these drugs. Using nanocarriers to protect drug cargo while it is in circulation can thus improve API pharmacokinetic and pharmacodynamics profiles. The augmented permeability of the tumor microenvironment (TME) neovasculature enables nanoparticles to preferentially accumulate in tumor tissue, thus promoting drug release. Nano formulation can improve the therapeutic window of the payload agent by diminishing systemic toxicity and augmenting drug efficacy (5). Nanoparticles present a viable method for intracellular delivery of proteins and small molecules, addressing challenges such as immunogenicity, brief plasma half-life, and inadequate penetration through biological membranes. Nanocarriers can surmount these limitations by chemically conjugating or encapsulating the protein or peptide. Hydrophobic ion pairing can alter the solubility of charged hydrophilic molecules, enhancing the encapsulation of peptides and nucleic acids within nanoparticle cores (6).

Since 1995, nanoparticles and other nanomedicines have been clinically validated as drug delivery systems, with DOXIL^®^ being the inaugural FDA-approved nanomedicine. The domain of nanomedicine has experienced considerable progress, marked by the emergence of numerous additional nanomedicines. The application for non-cancer-related diseases is notably significant, especially regarding vaccination delivery, exemplified by the development of various SARS-CoV-2 vaccines authorized in 2021 (7). More recently, especially concerning the treatment of solid tumors, the development of targeted therapies has fundamentally changed the design process of new drug modalities. Independent of increased delivery alone, these next-generation nanoparticles are more frequently multifunctional, where their surface can be functionalized while also entrapping therapeutics inside the nanoparticle, so generating more creative approaches in actively targeting tumor sites and eliciting biological effects with therapeutic usefulness (8). Engineered biopolymer nanoparticles have also gained increasing attention in the food science sector due to their unique ability to enhance food quality, safety, and shelf stability (9). Owing to their biocompatibility, biodegradability, and structural tunability, biopolymers such as chitosan, alginate, cellulose derivatives and starch-based materials can be engineered into nanoscale systems capable of encapsulating bioactive compounds, antioxidants, antimicrobials, and nutraceuticals (10, 11). At the nanoscale, these polymers exhibit improved barrier properties, higher surface area, and superior interaction with food matrices, enabling controlled release of active agents, inhibition of microbial growth, reduction of oxidation, and delay in quality deterioration (12). Moreover, biopolymer nanoparticles can protect sensitive compounds, such as essential oils, vitamins, and natural preservatives, from environmental degradation, thus enhancing their stability and functional performance throughout storage (13). As a result, engineering biopolymer-based nanostructures represents a promising strategy for safe and sustainable food preservation, aligning with the increasing global demand for minimally processed foods and clean-label technologies.

## 2 The evolution of nanoparticles in cancer therapy

### 2.1 First-generation vs. next-generation nanoparticles

Nanotechnology, now recognized as a general-purpose technology, is not a novel concept. First-generation nanoparticles are passive nanostructures. Their ability to enhance drug tolerability, circulation half-life, and efficacy has facilitated their clinical application as pharmacological drug delivery carriers. In 1986, Maeda et al. validated the selective accumulation of nanoparticles at tumor sites, known as “passive targeting,” establishing the enhanced permeability and retention (EPR) effect as the “gold standard” for the localization of nanomedicines in solid tumors. Due to their discontinuous distribution in tumors, which enhances the enhanced permeability and retention (EPR) effect, nanoparticles traverse cancerous cells more efficiently than surrounding normal tissue, exhibiting greater permeability. Moreover, the obstruction of lymphatic drainage at the tumor site results in prolonged retention of nanoparticles within the tumor tissue.

The EPR effect on nanoparticles such as Doxil and Caelyx is sanctioned; however, the execution of passive targeting is problematic due to its substantial dependence on tumor biology (14). The EPR effect is consistently heterogeneous, exhibiting significant variation among tumors and individuals. A mere fraction (< 1%) of nanoparticles typically accumulates at the tumor site, likely attributable to the various challenges previously outlined. Research is ongoing regarding the utilization of Vasodilator agents and vascular disruption to augment absorption and improve the enhanced permeability and retention (EPR) effect. Additionally, the EPR effect has been documented in numerous rodent studies; however, extensive validation in human patients remains lacking. Through control of the tumor microenvironment and modification of the physicochemical characteristics of nanoparticles, researchers have optimized the accumulation of several forms of nanoparticles at tumor sites in the past two decades. Furthermore, developed as next-generation NPs by immobilizing and guiding ligands on their surfaces to precisely target tumor cells along with their sustained release, and environmental responsiveness, are active targeting Nanomedicines (15). This approach aims to improve the specific recognition and phagocytosis of nanoparticles by tumor cells increasing the curative effect. Many studies have shown that ligand-directed active targeting modified nanoparticles can precisely raise drug accumulation in tumors. Active targeting nanoparticles passively extravasate from blood vessels into tumor tissue, necessitating further examination of the factors contributing to enhanced accumulation at the tumor site (16).

Recent advances in nanomedicine have led to the development of several next-generation nanoparticle platforms that integrate targeting capability with stimuli responsiveness and multifunctionality to improve therapeutic efficacy. Lipid nanoparticles (LNPs) have attracted considerable attention due to their high biocompatibility and ability to efficiently encapsulate nucleic acids and small-molecule drugs. These systems have been widely explored for siRNA, mRNA, and CRISPR-based cancer therapies, enabling targeted gene silencing and modulation of oncogenic signaling pathways (17, 18). Similarly, polymeric nanoparticles, including those composed of poly(lactic-co-glycolic acid) (PLGA), polyethylene glycol (PEG), and chitosan derivatives, offer tunable physicochemical properties and controlled drug release profiles, making them highly suitable for targeted delivery of chemotherapeutics and biomacromolecules (19). Another promising class is inorganic nanoparticles, such as gold nanoparticles (AuNPs), silica nanoparticles, and iron oxide nanoparticles, which possess unique optical, magnetic, and photothermal properties that enable applications in theranostics, photothermal therapy (PTT), and magnetic resonance imaging (MRI)-guided drug delivery (20, 21). In addition, stimuli-responsive nanoparticles, designed to respond to internal tumor cues such as acidic pH, elevated glutathione levels, or enzymatic activity, as well as external stimuli including light, ultrasound, and magnetic fields, have demonstrated enhanced tumor specificity and controlled drug release within the tumor microenvironment (22, 23). More recently, biomimetic nanoparticles, including cell membrane-coated nanoparticles and exosome-based nanocarriers, have emerged as innovative delivery systems capable of evading immune clearance and achieving improved tumor targeting through natural biological interactions (24). Together, these next-generation nanoplatforms represent a major step toward precision oncology, enabling simultaneous drug delivery, imaging, and tumor-specific therapeutic activation, thereby overcoming many limitations associated with conventional nanoparticle systems.

### 2.2 Key design principles of advanced nanoparticles

Tumor tissue is perfect for active targeting research since its environment is acidic and receptor overexpression exists. By changing particle surface targeting ligands, researchers have created methods to lower toxicity and increase therapeutic efficacy. Ligand affinity influences the targeting capacity of nanoparticles; high-affinity ligands enable cell attachment and endocytosis but induce too high cytotoxicity to normal cells. Researchers have selectively targeted tumor cell receptors using the multivalence of ligand binding, avoiding normal cell receptors. Bindings between particles and cell surface receptors were repeated using a computational model. Showing a “super-selective” ability, multivalent nanoparticles enhanced binding force at particular receptor concentration thresholds, promoting endocytosis. Utilizing a FAR-targeting nanocomposite, Hong et al. raised binding affinity by 2500–170,000 times over free folate molecules. Carlson created a bifunctional conjugate and multivalent binding platform on the membrane surface showing poor interaction with anti-Gal antibodies and great affinity for integrin receptors. Tumor treatment depends much on this focused design. The activity of the ligand determines the target of a substance; chemical coupling or electrostatic adsorption can change this activity. When fibrinogen hooks to gold nanoparticles, side effects including inflammation can result. As biomolecules can quickly gather protein shells around them, the introduction of nanoparticles disturbs the equilibrium of proteins in biological fluids. Affecting particle fate, the “Vroman effect” controls the adsorption and desorption dynamics of the protein corona. Parenthetically, the Vroman effect describes the dynamic and competitive adsorption of plasma proteins onto material surfaces when they come into contact with biological fluids. Initially, small and highly abundant proteins such as albumin rapidly adsorb to the nanoparticle surface due to their high mobility and concentration. Over time, these proteins are gradually displaced by larger proteins with higher surface affinity, including fibrinogen, immunoglobulins, and apolipoproteins. This sequential exchange results in the formation of a dynamic protein corona that continuously evolves depending on the physicochemical properties of the nanoparticle surface and the surrounding biological environment. The Vroman effect is particularly relevant in nanomedicine because the composition of the protein corona can significantly influence nanoparticle stability, biodistribution, cellular uptake, immune recognition, and overall therapeutic performance. Surface characteristics such as charge, hydrophobicity, and functionalization with polymers or targeting ligands can modulate protein adsorption patterns, thereby affecting the biological identity of nanoparticles *in vivo* (25, 26). Mostly concentrated in the liver and spleen, nanoparticles have adsorption modifying ligand structure, either hampered binding or obscured targeting ability. Moreover, affecting ligand structure are non-specific interactions between biological macromolecules and nanomaterials (27, 28).

Cross-linking agents such as carbodiimide promote the covalent attachment of ligands to particle surfaces, enhancing their biocompatibility and efficacy in associating nanoparticles with specific ligands. Nanoparticles can be conjugated via copper-free azide-alkyne cycloaddition, affinity interactions, or metal coordination. Researchers have examined ligand coupling sites and their influence on protein structure. Vitronectin, a potential active targeting ligand protein, has been recognized for its ability to target tumor cell αvβ3 integrin receptors. Targeted ligands, including proteins, polysaccharides, synthetic peptides, aptamers, and small molecules, are frequently employed in drug delivery systems. Human transferrin (Tf), a biodegradable iron-binding protein, has been utilized in diverse drug delivery systems. Polypeptides such as arginine-glycine-aspartate (RGD) peptides possess significant tumor-targeting efficacy and have been utilized in systemic anti-angiogenic therapy. Aptamers, which are single-stranded DNA or RNA oligonucleotides, serve as superior targeting ligands owing to their diminutive size, sensitivity, biodegradability, and non-immunogenic characteristics. Receptor targeting is essential for improving specificity, with accumulation at tumor sites reliant on the enhanced permeability and retention (EPR) effect. Ligand-modified nanoparticles bind to specific receptors and penetrate cells through receptor-mediated endocytosis. Nonetheless, off-target effects and efflux may occur as a result of cellular multidrug resistance proteins. Ligand-receptor-mediated endocytosis is a drug delivery mechanism that produces vesicle-coated particles to circumvent transporters and facilitate cellular entry. The rate of receptor-mediated uptake is affected by particle size, with larger particles demonstrating increased binding affinity. Cells possess multiple mechanisms for internalization and particle uptake, including clathrin-mediated endocytosis, caveolin-mediated endocytosis, fluid-phase endocytosis, lipid raft endocytosis, and bulk endocytosis (29). Receptor-mediated endocytosis is essential for accurate drug delivery, particularly for pharmaceuticals aimed at organelles. The cell membrane is a dynamic biomembrane characterized by heterogeneous receptor distribution and protein expression. Receptor-mediated endocytosis entails the infiltration of particles into cells to recycle their ligands, thereby diminishing the quantity of receptors on the membrane surface. The recycling rate of folate receptors differs among cancer tissues, with maximum efficiency attained when the dosing frequency is less than tumor FRs. The expression of membrane receptors is affected by both external and internal factors, resulting in the creation of artificial receptors. Nethi et al. engineered bone marrow mesenchymal stem cells to express azido-salicylic acid, resulting in functionalized nanoparticles that enhance retention duration at tumor locations. This approach efficiently inhibits tumor growth and prolongs survival in mouse models. Nonetheless, there is no conclusive evidence that artificial receptors promote particle endocytosis. Researchers devised a targeted delivery approach utilizing gold Nanorods, amplifying disease signals *in vivo* and enabling accurate nanoparticle aggregation at tumor locations (30, 31).

### 2.3 How next-generation nanoparticles address limitations of traditional drug delivery

The active targeting of tumor cells has been a focal point since Paul Ehrlich’s concept of the “magic bullet” in the early 1900s. Improvements in synthesis and formulation methods have facilitated active or site-specific targeting, especially in cancer therapy. Eleven antibody-drug conjugates (ADCs) have received approval, and next-generation nanoparticles are currently employed in clinical applications. ADCs can surmount cellular target resistance, offering a secondary treatment alternative for patients exhibiting developed resistance. The advancement of ADCs entails the utilization of stable linkers and site-specific conjugation techniques to improve efficacy and toxicity (32). The Drug-to-Antibody Ratio (DAR) quantifies the number of cytotoxic drug molecules per antibody, constraining the efficacy of Antibody-Drug Conjugates (ADCs) by diminishing effective internalization and necessitating the use of high-potency payloads (33). Investigations into antibody-drug conjugates (ADCs) emphasize less potent cytotoxic agents, including trastuzumab deruxtecan and sacituzumab govitecan. Conjugating targeting moieties such as antibodies to nanoparticle surfaces enables the development of a novel “magic bullet” therapy. This method enables tumor-specific antibodies to engage with antigens on cancerous cells, improving the DAR and facilitating elevated drug concentrations. Antibodies represent merely one class of agents for targeting nanomedicines, and additional research will investigate alternative targeting agents (34, 35).

Active targeting strategies primarily rely on the specific interaction between targeting ligands on nanoparticle surfaces and overexpressed receptors on tumor cells or tumor-associated tissues. Cancer cells frequently exhibit elevated levels of receptors such as the folate receptor, transferrin receptor, epidermal growth factor receptor (EGFR), human epidermal growth factor receptor-2 (HER2), and integrins, which can be exploited for receptor-mediated endocytosis. By decorating nanoparticles with ligands capable of recognizing these receptors, the internalization of therapeutic cargo can be significantly enhanced compared with passive accumulation alone. For instance, folic acid-functionalized nanoparticles have demonstrated improved uptake in ovarian and breast cancer cells due to the high expression of folate receptors, while transferrin-modified nanocarriers have been used to deliver chemotherapeutic agents and nucleic acids through transferrin receptor-mediated transport pathways (36, 37). These ligand-receptor interactions facilitate selective binding and intracellular trafficking, thereby increasing local drug concentration within malignant tissues while minimizing exposure to healthy cells.

Beyond antibodies, a wide variety of targeting ligands have been investigated to improve the specificity and versatility of nanomedicine platforms. These include peptides, aptamers, small molecules, and carbohydrates, each offering unique advantages in terms of size, stability, and production cost. Peptide ligands such as arginine-glycine-aspartic acid (RGD) motifs can selectively bind to integrins that are overexpressed on tumor endothelial cells, promoting enhanced tumor penetration and anti-angiogenic effects. Similarly, nucleic acid-based aptamers have gained attention because of their high affinity, low immunogenicity, and ease of chemical synthesis, allowing them to function as antibody-like targeting agents in nanoparticle systems (36, 38). Carbohydrate-based ligands are also increasingly explored for targeting lectin receptors on tumor cells, providing additional opportunities for designing polysaccharide-based nanocarriers. Collectively, these targeting strategies broaden the scope of active nanomedicine design beyond traditional antibody-mediated approaches.

Recent developments have further integrated multifunctional targeting mechanisms into next-generation nanocarriers to overcome biological barriers associated with tumor heterogeneity and the complex tumor microenvironment. For example, dual-targeting nanoparticles incorporate two distinct ligands to simultaneously recognize tumor cells and tumor vasculature, thereby improving accumulation and penetration within solid tumors. Additionally, stimuli-responsive targeting systems have been engineered to release therapeutic payloads selectively in response to tumor-specific conditions such as acidic pH, hypoxia, or elevated enzymatic activity. These strategies enhance intracellular drug delivery while reducing premature drug leakage in systemic circulation (18, 37). Advances in surface engineering, including polyethylene glycol (PEG) modification and biomimetic coatings derived from cell membranes, have also improved nanoparticle stability and immune evasion, enabling longer circulation times and increased opportunities for receptor-mediated targeting. Together, these innovations represent significant progress toward realizing Ehrlich’s vision of a “magic bullet,” in which nanomedicines selectively recognize and eradicate malignant cells while sparing normal tissues.

## 3 Types of next-generation nanoparticles for cancer therapy

### 3.1 Lipid-based nanoparticles: liposomes, solid lipid nanoparticles, nanostructured lipid carriers

**FIGURE 1:**
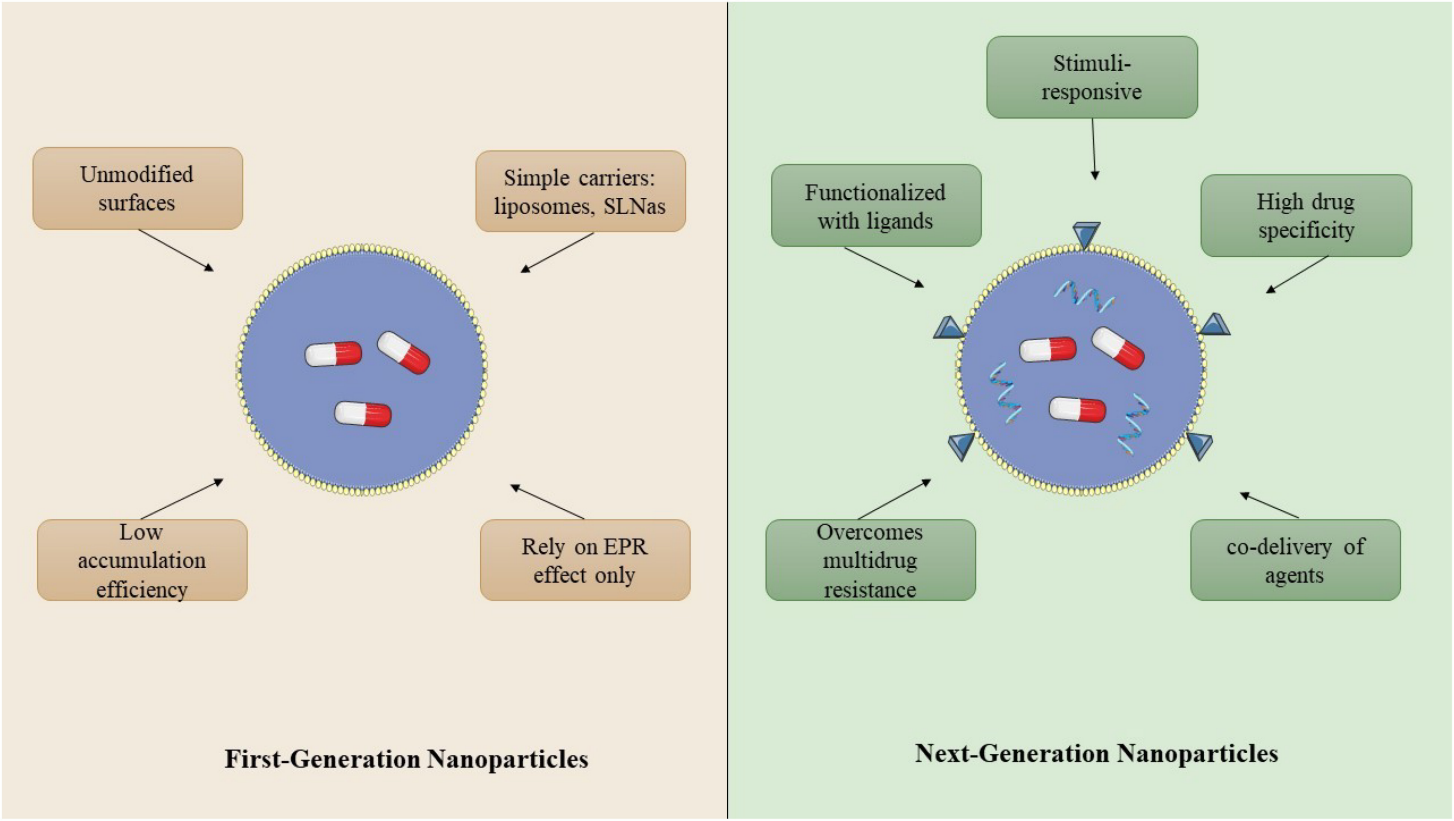
Illustration of the transition from first-generation nanoparticles, which rely solely on passive targeting mechanisms such as the enhanced permeability and retention (EPR) effect, to next-generation nanoparticles equipped with active targeting ligands and stimuli-responsive capabilities. While first-generation systems suffer from limited specificity and poor tumor accumulation, advanced designs incorporate surface functionalization, environmental responsiveness (e.g., pH, redox), and multifunctional payloads to enhance therapeutic efficacy and precision in cancer treatment.

Lipid-based nanoparticles (LNPs) are multifunctional nanocarriers employed in medical research and pharmacology for encapsulating therapeutic agents, ensuring drug protection, enhancing solubility, facilitating targeted delivery, and optimizing bio distribution (Figure 1) (39). Liposomes, self-assembling nanoscale lipid vesicles, serve as versatile drug delivery systems for therapeutic agents, imaging compounds, nucleic acids, and proteins. MD simulations elucidate liposome properties, whereas computational prodrug design enhances drug encapsulation and release. FDA has approved liposome-based pharmaceuticals such as Doxil for medical applications. Nanoemulsions are spherical liquid droplets comprising oil and water phases that encapsulate hydrophobic or hydrophilic compounds. Emulsifiers stabilize droplets by preventing aggregation via electrostatic repulsion, steric hindrance, and interactions from thermal fluctuations (40).

Nanoemulsions are colloidal formulations employed to encapsulate hydrophobic pharmaceuticals, thereby mitigating adverse effects. They are frequently utilized in topical drug delivery and the food sector for flavoring, coloring, nutraceutical, and preservative functions. Nonetheless, they demonstrate thermodynamic instability and are vulnerable to environmental factors. MD simulations are employed to investigate the dynamic behavior, interactions, and stability of Nanoemulsions under diverse conditions. These simulations facilitate the prediction of interactions between surfactants and hydrophobic pharmaceuticals to enhance solubilization and stability (41).

Solid lipid nanoparticles (SLNs) are spherical entities characterized by a solid lipid core, measuring between 50 and 100 nm in diameter. They may encompass active constituents, including antioxidants, anticancer compounds, nucleic acids, antibiotics, cytokines, and hydrophobic pharmaceuticals, and have been investigated in clinical trials. Molecular dynamics simulations have been employed to investigate the molecular properties and behavior of solid lipid nanoparticles, revealing their stability, structural alterations, and drug encapsulation efficiency. SLNs represent a promising drug delivery system owing to their technical advantages, which encompass drug protection, stability, scalability, sterilization capabilities, and biodegradability. Solid lipid nanoparticles (SLNs) encounter obstacles such as restricted drug loading efficiency, abbreviated shelf life, low loading capacity, polydispersity, and elevated operational temperature. Researchers have engineered nanostructured lipid carriers (NLCs) to address these constraints. These carriers integrate solid and liquid lipids, augmenting drug loading capacity and release kinetics. The ratio of solid lipid, liquid oil, and surfactant is essential for the efficiency of therapeutic agent entrapment. Nanocarriers can facilitate gene therapy and personalized medicine by regulating gene expression and transporting therapeutic proteins. MD simulations indicate that elevating the temperature to 358 K enhances the stability of NLC and promotes component compaction (42). Nanoparticles (NLCs) provide advantages such as augmented drug-loading capacity, controlled release, and improved stability for drug delivery. They possess biocompatibility and biodegradability, rendering them optimal carriers for diverse pharmaceuticals, gene therapy, and chemotherapy, as well as applications in the food and cosmetic industries. Lipid polymer hybrid nanoparticles (LPNs) integrate lipids and polymers for biomedical applications, facilitating sustained release and targeted delivery to particular cells or tissues. Density functional theory (DFT) simulations indicate strong interactions between polymers and DOX, implying enhanced oral bioavailability. Lipid-polymer nanoparticles (LPNs) are progressively acknowledged as a feasible substitute for traditional drug delivery systems owing to their versatility in diverse biomedical applications. These organic nanoparticles can be produced through physical, chemical, or biological techniques. Physical methods entail the reduction of larger materials in size, whereas chemical methods synthesize nanoparticles from molecular or atomic precursors. Biological methods utilize natural sources such as algae or plants for synthesis. Physical techniques encompass laser ablation, evaporation-condensation, sputtering, and high-energy ball milling. Chemical methodologies encompass sol-gel, microemulsion, precipitation, chemical vapor deposition (CVD), hydrothermal synthesis, and sonochemical synthesis. Biological techniques encompass algae-mediated synthesis, green synthesis, microwave-assisted synthesis, pyrolysis, emulsion-based methods, and self-assembly. Microwave-assisted synthesis employs microwave radiation to expedite chemical reactions, whereas pyrolysis involves the thermal decomposition of materials. Emulsion-based techniques employ methods such as emulsion polymerization or solvent evaporation, while self-assembly enables molecules or nanoparticles to autonomously arrange into structured formations (43). Nonetheless, challenges such as polymer toxicity and the consistency of particle size and shape remain. Table 1 comparatively summarizes lipid-based nanoparticles and their key characteristics.

**TABLE 1:** Comparative summary of lipid-based nanoparticles and their key characteristics.

Nanoparticle type	Advantages	Limitations	Stability considerations	Translational challenges
Liposomes^1^	•Biocompatible and biodegradable •Capable of loading both hydrophilic and hydrophobic drugs •FDA-approved formulations (e.g., Doxil) •Tunable surface for targeted delivery	•High production cost •Prone to leakage of encapsulated drugs •Limited shelf stability	•Sensitive to oxidation, hydrolysis, and pH •Require stabilizers or PEGylation to maintain integrity	•Scaling up remains expensive •Batch-to-batch variability •Cold-chain storage often required
Nanoemulsions	•High solubilization of hydrophobic drugs •Transparent and suitable for food applications •Easy to manufacture at scale	•Thermodynamically unstable •Prone to creaming, coalescence, and Ostwald ripening	•Strongly affected by pH, temperature, ionic strength •Require emulsifiers for stabilization	Regulatory concerns in foods Need for GRAS surfactants •Short shelf life limits commercial deployment
Solid lipid nanoparticles (SLNs)	•Solid core enhances drug protection •Good biocompatibility and biodegradability Scalable and sterilizable •Strong stability at room temperature	•Low drug-loading capacity •Risk of drug expulsion during storage •Polymorphic transitions affecting quality	•Crystallization behavior impacts stability Sensitive to storage temperature	Reproducibility and long-term stability •Difficulty maintaining uniform particle size •Limited capacity for hydrophilic drugs
Nanostructured lipid carriers (NLCs)	•Higher drug-loading capacity than SLNs •Reduced drug expulsion •Flexible release profiles Suitable for pharmaceuticals, cosmetics, and foods	•More complex formulation than SLNs •Requires careful optimization of lipid ratios	•Mixed solid-liquid matrix improves physical stability •Still sensitive to thermal fluctuations	•Need for precise control of lipid composition •Challenges ensuring long-term stability in commercial formulations
Lipid-polymer hybrid nanoparticles (LPNs)	•Combine structural integrity of polymers with biocompatibility of lipids •Suitable for sustained release and targeting •Improved bioavailability (supported by DFT studies)	•Polymer toxicity concerns •Complexity of synthesis •Potential for inconsistent morphology	•Stability depends on polymer-lipid interactions •Sensitive to environmental stressors	•More complicated manufacturing and regulatory approval •Difficulty achieving uniformity at industrial scale

^1^This table has indeed summarized the information provided in section 3.1., and thus no reference(s) have been mentioned in the table.

### 3.2 Polymeric nanoparticles: PLGA, PEGylated nanoparticles, dendrimers

Polymers, derived from monomers, can exhibit diversity and non-toxicity. Biodegradable and biocompatible polymeric nanoparticles are composed of natural monomers. They are frequently utilized in drug delivery, with carbohydrates and proteins as subdivisions. Subsequent discourse regarding these nanoparticles will ensue (44).

#### 3.2.1 Carbohydrate-based nanoparticles

Polysaccharides, sourced from plants, animals, and microorganisms, serve as drug delivery nanoparticles owing to their biocompatibility, non-toxicity, biodegradability, substantial drug loading capacity, and targeted release properties. Chitosan, a polysaccharide, is synthesized from chitin via partial N-deacetylation. The properties fluctuate based on the preparation method, with emulsion techniques providing superior particle size regulation and efficacy in incorporating both hydrophilic and hydrophobic drugs. Ion gelation techniques enhance the biocompatibility of nanoparticles and diminish the presence of undesirable particles. The properties of chitosan are affected by its origin and processing method, with degradability diminishing as deacetylation increases and toxicity reducing as molecular weight decreases. It is utilized in cosmetics, pharmaceuticals, and medical procedures such as tissue regeneration. Nonetheless, chitosan exhibits inadequate mechanical properties, insufficient thermal stability, limited ductility, elevated hydrophobicity, and a rapid degradation rate. To enhance its properties, it is frequently cross-linked with ligands and natural polymers. The polycationic characteristics of chitosan enhance its solubility and prolong its interaction with mucous membranes. Chitosan nanoparticles are advantageous for medications administered orally, transdermally, and intranasally, as well as via vaginal, buccal, and cutaneous routes. They exist in diverse forms, including nanospheres, nanofibers, and nano-capsules (45).

Alginate, a gelling polymer derived from brown marine algae, is utilized for drug delivery owing to its biodegradability and insolubility in organic solvents. Their nanoparticles are utilized for pharmaceuticals and biotechnological compounds. Starch, a biopolymer derived from staple foods, is utilized for topical, parenteral, and oral drug delivery. Both materials are employed in cancer treatment owing to their capacity to diminish side effects and enhance drug accumulation in tumor cells (46). Dextran nanoparticles are natural polysaccharides utilized in medical and pharmaceutical domains owing to their biocompatibility, non-immunogenicity, and biodegradability. They augment drug solubility, extend drug retention duration, and enhance bioavailability. Dextran nanoparticles can be synthesized through self-assembly, emulsification, co-precipitation, and spray-drying techniques. They have been utilized in cancer therapy to improve water solubility, prolong circulation time in the bloodstream, and facilitate permeation to malignant cells. Dextran-coated iron oxide nanoparticles have been employed to enhance the visibility of cells and tissues. Dextran-based nanoparticles have been utilized in insulin formulations, yielding a superior hypoglycemic effect relative to traditional insulin preparations. Ibuprofen has been encapsulated in dextran nanoparticles to alleviate the side effects of methylprednisolone in the treatment of spinal cord injuries (47). Protein-based nanoparticles are biodegradable, metabolizable, and readily modifiable. They are utilized in optics, radiation, catalysis, ultrasound, magnetism, gene therapies, and oncological treatment. They are constructed utilizing proteins such as albumin, gelatin, and ferritin for pharmaceutical delivery. Albumin nanoparticles are non-toxic, biodegradable, and readily available. They augment the therapeutic effects of medications and can be altered with diverse substances. Gelatin nanoparticles are biopolymers characterized by high biodegradability, low immunogenicity, and cost-effectiveness. They are utilized in the treatment of cancer, HIV infection, tuberculosis, and bacterial infections (48). Functionalized nanoparticles have been employed as DNA carriers in neoplastic tissues, for the treatment of Leishmaniasis, and in genetic engineering. Ferritin nanoparticles exhibit effective drug-loading capabilities owing to their hollow cavity, low toxicity, minimal immunogenicity, high biodegradability, and excellent biocompatibility. They are directed toward tumor cells and utilized in tumor imaging, immunotherapy, and vaccines (49).

### 3.3 Inorganic nanoparticles: gold, silica, quantum dots, iron oxide nanoparticles

Inorganic nanoparticles, including iron oxide, quantum dots, and gold, are extensively utilized in drug and gene delivery owing to their hydrophilicity, non-toxicity, biocompatibility, and stability. Inorganic nanoparticles can be produced through top-down or bottom-up methodologies. Top-down methods entail the fragmentation of larger materials into nanoparticles, whereas bottom-up methods synthesize nanoparticles from atomic or molecular precursors. Common methodologies encompass ball milling, lithography, sol-gel synthesis, chemical vapor deposition, microemulsion techniques, hydrothermal synthesis, green chemistry strategies, inert gas condensation, precipitation, microwave-assisted synthesis, ultrasonic-assisted synthesis, and biotemplated synthesis. Bottom-up methodologies encompass chemical vapor deposition, microemulsion synthesis, hydrothermal synthesis, green chemistry techniques, inert gas condensation, precipitation, microwave-assisted synthesis, ultrasonic-assisted synthesis, and biotemplated synthesis. Gas-to-solid techniques, liquid-phase synthesis, precipitation methods, *in situ* characterization, and microfluidic devices are employed in nanoparticle synthesis. In summary, inorganic nanoparticles can be synthesized through mechanical techniques such as ball milling, lithography, sol-gel synthesis, chemical vapor deposition, microemulsion methods, hydrothermal synthesis, green chemistry approaches, gas-to-solid methods, liquid-phase synthesis, precipitation methods, *in situ* characterization, and microfluidic devices (47, 50).

#### 3.3.1 Iron oxide nanoparticles

Iron oxide nanoparticles exhibit exceptional biocompatibility, non-toxicity, stability, and biodegradability, making them extensively utilized in electronics, biotechnology, biomedicine, and energy owing to their responsiveness to magnetic fields. They have proven effective in cancer therapy, gene delivery, and tissue engineering, exhibiting improved targetability when coated with mannan (51).

#### 3.3.2 Quantum dots

Quantum dots, created in 1981, are utilized in photodetectors, LED manufacturing, drug delivery systems, and imaging technologies. They exhibit low toxicity, stability, and biocompatibility. They can modulate doxorubicin release and diminish cancer cell size. They are utilized in bio-imaging to enhance the penetration of visible light in biological tissues (52).

#### 3.3.3 Gold nanoparticles

Gold nanoparticles exhibit distinctive characteristics suitable for drug delivery, imaging, and tumor treatment. They can convey vaccines, proteins, and nucleotides through covalent, ionic, or physical bonding. They can additionally be altered with biomolecules such as nucleic acids, antibodies, and peptides. Their diminutive dimensions, adaptable surfaces, and varied morphologies render them advantageous for vaccine administration (53).

### 3.4 Hybrid and stimuli-responsive nanoparticles: pH-responsive, temperature-sensitive, and redox-activated carriers

Hybrid nanoparticles comprise two distinct nanoparticles, frequently integrating or surpassing the limitations of an individual component. They may be organic, inorganic, or a hybrid of the two. There are three categories: organic hybrids, inorganic hybrids, and organic-inorganic hybrids. A variety of hybrid nanoparticles have been employed for drug delivery, including lipid-polymer, metal-organic, and porous silica-based types.

#### 3.4.1 Lipid-polymer hybrid nanoparticles

A single-step synthesis technique has facilitated the transport of genetic materials and therapeutic agents via lipid-polymer hybrid nanoparticles. These nanoparticles have been utilized to administer various chemotherapeutic agents, actively targeted drugs, and target cells via antibodies, aptamers, and transferrin. Gold nanocrystals and quantum dots have been incorporated into PLGA as imaging agents, demonstrating their efficacy and appropriateness for *in vitro* studies (54).

#### 3.4.2 Metal-organic hybrid nanoparticles

Metal-organic frameworks (MOFs) are hybrid nanoparticles characterized by adjustable properties, including responsiveness to pH, light, and temperature. These multifunctional particles are employed in drug delivery, anticancer treatment, antiviral treatment, theranostics, bioimaging, and gene therapy. MOFs exhibit diverse morphologies, contingent upon synthesis parameters, organic linkers, solvents, and metal ions. The synthesis methods encompass microwave-assisted, ultrasonic, and hydrothermal techniques. The amalgamation of MOFs with quantum dots, optical imaging, platinum, and ZIF-C has demonstrated enhanced sensitivity, superior image quality, reduced toxicity, and improved drug delivery. MOFs are recognized for their superior biocompatibility, rendering them advantageous in diverse medical applications. They created through various methods, including liquid-phase synthesis, mechanochemical and electrochemical approaches. Liquid-phase synthesis involves dissolved metal salts and organic linkers in a solvent, while solid-state synthesis uses mechanical energy to initiate reactions. Electrochemical synthesis generates metal ions *in situ*, forming MOFs on electrode surfaces. Other techniques include microwave-assisted synthesis, sonochemical synthesis, diffusion method, post-synthetic modification, and MOF hybrids. Solvothermal/hydrothermal synthesis involves dissolved metal salts and organic linkers in a solvent, heated under pressure in an autoclave. Solid-state synthesis uses mechanical energy to initiate reactions, while electrochemical synthesis generates metal ions *in situ*. Microwave-assisted synthesis accelerates synthesis, while sonochemical synthesis uses ultrasound for crystal formation. Post-synthetic modification introduces new functionalities or improves properties. MOF hybrids can be created by combining MOFs with other materials, using methods like *in situ* growth, post-synthetic modification, or physical mixing (55).

#### 3.4.3 Porous silica-based hybrid nanoparticles

Silica-based hybrid nanoparticles, created by combining silica with various materials, exhibit non-toxicity, stability, and biocompatibility, rendering them appropriate for cancer therapy, drug delivery, and diagnostic applications. Their extensive surface area and susceptibility to functional groups render them adaptable for cellular internalization. Silica hybrid materials can be synthesized through multiple techniques, including the sol-gel method, grafting, co-condensation (*in situ* grafting), and the application of periodic mesoporous silica. The sol-gel technique entails the hydrolysis and condensation of silica precursors, like tetraethyl orthosilicate (TEOS), within a solution, frequently accompanied by an organic component. This method facilitates the synthesis of diverse silica-based hybrid materials, encompassing polymers, biomolecules, and various organic moieties. The grafting-to method entails the covalent attachment of pre-formed polymer chains to the surfaces of pre-formed silica nanoparticles (SNPs), facilitating the precise incorporation of specific organic functionalities onto the silica surface. Co-condensation (*in situ* grafting) entails the concurrent hydrolysis and condensation of silica and organic precursors, resulting in hybrid materials with the organic component embedded within the silica matrix. Alternative techniques for synthesizing silica nanoparticles encompass microemulsions, hydrothermal synthesis, and layer-by-layer assembly. These methods facilitate the integration of diverse organic constituents into the silica matrix, resulting in materials with customized properties (56).

#### 3.4.4 Stimuli-responsive nanoparticles: external and internal stimuli

##### 3.4.4.1 External stimuli: thermal, magnetic, ultrasound, and light stimuli

External stimuli such as thermal, magnetic, electronic, ultrasound, and light can influence the behavior of nanocarriers in biological systems, facilitating controlled release, intracellular drug delivery, and cancer treatment. Nonetheless, these stimuli remain unattainable for metastatic lesions (57). Ultrasound, a high-frequency acoustic wave, can be utilized to regulate drug release at pathological locations, such as tumors. It can be modified for various applications, including imaging at low frequencies (< 20 kHz) or disrupting Nanocarriers to release cargo or improve cancer cell membrane permeability at high frequencies (> 20 kHz). Ultrasound-sensitive nanocarriers have been engineered for ultrasound imaging, drug delivery, and cancer theranostics. These nanocarriers can encapsulate gaseous or contrast agents, augment tumor accumulation, and enhance drug delivery, although their dimensions may limit infiltration into tumor tissues (58). Temperature-sensitive nanocarriers are employed for drug delivery and oncological therapy. These materials exhibit stability at standard temperatures but are susceptible to elevated temperatures, resulting in substantial alterations to their properties. Diverse formulations encompass liposomes, polymeric micelles, nanocomposites, nanocapsules, nanogels, and vesicles. The materials, including poly (N-isopropyl acrylamide), poly (N-vinyl isobutyrate), poly(2-oxazoline), and poly [2-(2-methoxy ethoxy) ethyl methacrylate] (PMEOMA), can exhibit variations in physicochemical properties in response to temperature fluctuations. The integration of thermally unstable materials can also attain thermal sensitivity. Thermal-sensitive nanocarriers facilitate gene and drug delivery through the utilization of thermoresponsive polymeric materials that transition from hydrophilic to hydrophobic states. These materials can generate siRNAsomes, facilitate protein folding, and incorporate hydrophobic anticancer agents. They may also be utilized for the controlled release of cargo in pathological areas exhibiting local hyperemia. Doxorubicin can be released from lipid-peptide vesicles in response to mild hyperemia, whereas Nile Red and doxorubicin can be released from polymeric micelles in response to thermal stimuli. Nevertheless, there exists a limited selection of thermally sensitive materials, and certain materials fall outside the parameters of biological systems or cannot be adjusted to an alternative temperature. Certain thermal-responsive nanocarriers are constructed from non-biodegradable polymers, posing challenges for clinical translation. Consequently, the advancement of biodegradable and thermally responsive materials represents a prospective trajectory. The aggregation of nanocarriers in tumors is essential for facilitating targeted thermal-triggered drug release and treatment (59).

Magnetic-responsive nanocarriers have been engineered to specifically target tumors and induce localized hyperthermia for drug release and tumor ablation. The nanocarriers comprise magnetic nanoparticles, liposomes, superparamagnetic iron-oxide nanoparticles (SPIONs), polymeric micelles, albumin nanocapsules, magnetic nanocarriers, and magnetic nanogels. They utilize magnetic materials such as iron oxide nanoparticles, graphene/Au/Fe_3_O_4_ hybrids, and various other magnetic nanomaterials. Magnetic nanocarriers are applicable for tumor imaging, and delivery of anticancer agents, plasmids, antibodies, and photosensitizers. They may serve as either passive or active targets for cancer cells. The interplay between magnetic nanocarriers and magnetic fields facilitates the targeted accumulation of bioactive compounds within tumors. The hyperthermia induced by an alternating magnetic field facilitates the targeted release of heat shock protein inhibitors, thereby promoting apoptosis without resistance for effective tumor ablation. These nanocarriers possess potential for the treatment of metastatic tumors and tumor theranostics. Superparamagnetic materials within these nanocarriers may be utilized for targeted tumor therapy (60). Light-responsive nanocarriers are engineered to specifically target biological systems, such as cancer cells and tumors, by adjusting wavelength, intensity, and the area of impact. These nanocarriers can alter molecular conformation, cleave light-sensitive chemical bonds, initiate therapeutic release, facilitate light-activated imaging, produce singlet oxygen, generate reactive oxygen species, and induce photothermal effects for tumor ablation. They can be synthesized or constructed by responding to light, modifying hydrophilic-hydrophobic balance, or experiencing structural transformation. Recently, light-sensitive nanoparticles were synthesized utilizing DSPE-PEG to incorporate spiropyran under visible or dark conditions, indicating the potential for UV-Vis-activated drug release and tumor ablation (61).

##### 3.4.4.2 Internal stimuli: pH, redox, and hypoxia

The research investigates the influence of biological factors within the tumor microenvironment, including enzymes, ATP, low pH, redox potential, and hypoxia, as catalysts for regulated drug release, endosome/lysosome evasion, prodrug activation, and tumor-targeted imaging and therapy. It also addresses recent advancements in nanocarriers that respond to internal stimuli for tumor theranostics. pH-responsive nanocarriers have been widely utilized in oncological therapies owing to the acidic pH present within cancer cell organelles and the tumor microenvironment. pH-sensitive nanocarriers have been utilized for diverse applications, including imaging, intracellular drug delivery, charge conversion, and controlled drug release within the tumor microenvironment. These nanocarriers consist of pH-sensitive polymers and can be triggered by intracellular pH for drug release within cancer cells or the tumor microenvironment. They can also facilitate intracellular drug delivery, allowing for the transport of antibodies, proteins, siRNA, and DNA while improving tumor accumulation. PDNA-loaded nanocarriers have been designed for targeted gene therapy in oncology, whereas pH-sensitive nanoparticles facilitate endocytosis, intracellular drug release, and cancer-specific theranostics. These nanocarriers may also serve as imaging probes, facilitate fluorescence imaging, and enable neutron capture therapy (62). Hypoxia, a condition characterized by diminished oxygen levels in tumors, is a critical factor in cancer progression and resistance to standard therapies. In response to this issue, numerous nanocarriers have been engineered for drug delivery to hypoxic tumors, encompassing hypoxia-responsive and pH-sensitive variants. These nanocarriers enable molecular imaging, metastasis, and the transport of hypoxia-activatable prodrugs. Nonetheless, obstacles such as regulating the tumor microenvironment, enhancing drug infiltration, and elevating oxygen levels persist (Table 2) (63).

**TABLE 2:** pH and redox-responsive Nano substances for the release of anti-cancer drugs.

Nano substance composition	Mode of operation	Cargo	References
Polyethylene glycol, hyaluronic acid	Calcium phosphate dissolution	Doxorubicin	(66)
Polyethylene glycol, polycaprolactone	beta-carboxylic amides link hydrolyzation	Doxorubicin	(67)
Au, mesoporous silica	Host-guest interaction system release of the hydrophilic Fc +	Doxorubicin	(68)
Zirconia	Molecular switching	Doxorubicin	(69)
Mesoporous silica	Disulfide link destabilization	Doxorubicin	(70)
Gold	pH stimuli responsive coating	Epirubicin	(71)
Poly-L-Leucine, poly-L-Lysine	beta-carboxylic amides link hydrolyzation	Doxorubicin	(66)
Polylactic glycolic acid	Acidity triggered rational membrane destabilization	Doxorubicin	(72)
Mesoporous silica	Disulfide link destabilization	Doxorubicin	(73)
Polydopamine	Disulfide link destabilization	Doxorubicin	(74)
Poly-l-Arginine, polydopamine	Bis-norbornene as acid-labile linker destabilization	Doxorubicin	(75)
Au, mesoporous silica	Host-guest interaction system release of the hydrophilic Fc +	Doxorubicin	(73)
Poly (3-caprolactone), poly (N, N-dimethylamino-2-ethyl methacrylate)	Disulfide link destabilization	Doxorubicin	(76)
Hyaluronic acid	pH stimuli sensitive lipids	Doxorubicin	(77)
Phospholipid, polyurethane	Acetal link destabilization	Doxorubicin	(67)

Redox-responsive nanocarriers, such as nanocapsules, silica nanoparticles, and polymer-drug conjugates, are employed for drug delivery owing to their reduction potentials and tumor-targeting abilities. These nanocarriers can trigger cargo release within cancer cells, promote disassociation, and enable regulated dissociation. Disulfide bonds serve as a redox-sensitive connector in nanocarriers, remaining stable in the bloodstream yet readily cleaved in cancer cells owing to elevated glutathione concentrations. Additional redox-responsive moieties, such as diselenides, thioethers, and thiol groups, may also be integrated. Polymeric micelles, liposomes, and nanogels can be engineered with disulfide bonds to respond to redox environments, facilitating cargo release within cancer cells, while liposomes can be altered with redox-sensitive linkers for drug delivery (64). They can also surmount barriers such as siRNA and sodium borocaptate, enhancing the intracellular transport of bioactive compounds. Staggered lamellae demonstrate optimal efficiency in cellular internalization. These nanocarriers possess considerable potential for the treatment of hypoxic tumors (Table 2) (65).

##### 3.4.4.3 Enzyme-responsive nano carriers

Enzymes are essential in biological reactions, and their unregulated expression in neoplastic conditions can trigger enzyme-responsive drug delivery. Enzyme-sensitive nanocarriers have been engineered for targeted drug delivery in tumors and cancer cells, facilitating prodrug activation, morphological modification, and physical disruption. These nanocarriers can respond to increased enzyme concentrations in the tumor microenvironment and cancer cells. Enzyme-sensitive nanocarriers possess considerable potential in the diagnosis and treatment of primary and metastatic tumors (78). PEGylated nanoparticles featuring MMP-degradable linkers facilitate the attachment of drugs, enabling their release upon cleavage by MMPs. Nanocarriers responsive to MMP-2 and MMP-9 utilize peptide sequences for targeted drug release at tumor locations. Manganese nanoparticles encapsulated with collagen-IV peptides are employed for drug delivery and MRI-based tumor visualization. Cathepsin B-sensitive peptide linkers, hyaluronic acid-based nanoparticles, ATP-responsive liposomes and micelles, and 4-Phenylbutyric acid-modified hyaluronic acid-based nanoparticles are engineered to release pharmaceuticals in reaction to enzyme activity, enzymatic degradation by hyaluronidase, elevated ATP levels in metabolically active tumor cells, and esterase activity in 4-PBA-modified hyaluronic acid-based nanoparticles (79).

### 3.5 Biodegradable and bioinspired nanocarriers: cell-membrane-coated nanoparticles, exosome-mimicking systems

Researchers are employing nanotechnology to develop versatile and efficacious nanomedicines that replicate biological characteristics, surmounting biological obstacles. These bioinspired nanomedicines exhibit biocompatibility, biodegradability, and enhanced circulation efficiency for payload delivery. They are biocompatible, biodegradable, and possess enhanced capacity for circulation to facilitate therapeutic enhancement. The domain of drug delivery optimization has expanded considerably in recent decades, marked by the advancement of nano-sized drug delivery systems (NDDSs). NDDSs are engineered to transport and release pharmaceuticals at targeted anatomical locations, enhancing efficacy and minimizing adverse effects, utilizing materials such as liposomes, micelles, nanoparticles, dendrimers, and nanocrystals. Biomimetic nanomedicines derived from natural cells provide benefits over synthetic materials due to their superior biocompatibility and multifunctionality, addressing certain limitations of synthetic alternatives (80).

#### 3.5.1 Cell-membrane-camouflaged NPs

Nanomedicines camouflaged with cell membranes maintain the physicochemical characteristics of synthetic materials while acquiring biological functions from source cells, owing to the integration of membrane compositions with diverse surface molecules. Conventional chemical modifications are challenging to duplicate these surface characteristics and intricate biological functions. Numerous techniques have been devised to fabricate membrane-cloaked nanomaterials utilizing diverse membrane types. The synthesis of nanoparticles entails the isolation of the cell membrane, the preparation of a core material, and the fusion of the membrane with the core. Cells are lysed to extract and purify the membrane. Methods such as extrusion, sonication, or electroporation are employed to produce the final camouflaged nanoparticle. Complex biochemical procedures, such as hypotonic lysis and ultrasonication, are required for nucleated cells. The synthesized cores may be enveloped with the membrane through physical extrusion; however, laboratory productivity has been suboptimal, rendering sonication more efficacious. Nanomedicines disguised with cell membranes possess the potential to surmount biological barriers for targeted delivery. The integrity of the cell membrane coating on nanoparticles is a crucial criterion for assessing their efficacy (81, 82).

#### 3.5.2 Whole cell as drug carrier

The living cell presents a complete system for biological applications, facilitating the encapsulation of therapeutic agents and drug delivery across diverse cell types, while the external membrane ensures strong adhesion (83).

#### 3.5.3 Red blood cells

Red blood cells (RBCs) possess a distinctive morphology, an optimal surface area-to-volume ratio, and a flexible cytoskeleton, rendering them resilient to lysis and fracture. RBCs are employed in numerous applications, such as the treatment of leukemia, prolonging the half-life of tramadol, and administering medications like steroids, thrombolytic agents, and antigens to influence immune responses or target specific tissues. They facilitate extended drug delivery owing to their significant drug-loading capacity and superior biocompatibility. Pharmaceutical nanocarriers originating from red blood cells have been investigated for targeted cancer treatment. Methods such as osmotic lysis and non-invasive cell-penetrating peptide-mediated internalization have been employed to encapsulate pharmaceuticals within red blood cells. Nevertheless, these techniques may inflict irreversible harm on the cell’s structural integrity and impede drug diffusion. RBC-based Direct Drug Delivery Systems (DDSs) seek to improve circulation time, drug-loading capacity, and biocompatibility (84). They can be utilized for diverse therapies, encompassing the encapsulation of enzymes such as L-asparaginase, dexamethasone, and thymidine phosphorylase, surface modification with targeting agents like biotin or antibodies, and RBC-inspired methodologies such as hitchhiking with nanocarriers. These strategies utilize the extended circulation duration of RBCs and their immune clearance, while enabling the expression of therapeutic proteins or enzymes, potentially addressing metabolic disorders or compensating for deficient enzymes (85).

#### 3.5.4 Macrophages and monocytes

Macrophages are essential immune cells for targeted drug delivery in cancer therapy. Therapeutic nanoparticles can be internalized by macrophages via phagocytosis, enhancing therapeutic efficacy. Researchers have devised an *in vivo* internalization technique for drug encapsulation based on receptor-mediated phagocytosis. Intelligent nanodrugs promote the transpolarization of M2 macrophages to M1, thereby inhibiting tumor growth and metastasis. Nanoparticles can target the CSF1R pathway, essential for M2 macrophage survival, to eradicate M2-like macrophages and enhance anti-tumor immunity. NPs administering miR-125a, a non-coding RNA with tumor-suppressive properties, can counteract immune suppression by reprograming M2 macrophages to M1 and inhibiting tumor proliferation. Nanomaterials enhanced with bacterial derivatives, such as DOX@Bio-Bac or MNPs-MPLA-siRNA, can facilitate the polarization of M2 macrophages to M1 and trigger apoptosis in cancer cells. Nanozymes, such as Cu-doped polypyrrole nanozymes (CuPP), can elevate oxygen concentrations in the tumor microenvironment, ameliorating hypoxia and facilitating the transformation of M2 macrophages to M1, thereby enhancing the immune response (86). Tumor-infiltrating macrophages possess intrinsic phenotypic plasticity, allowing them to adapt to tumor mesenchymal stem cells. Targeting Ly-6Chi monocytes in the circulation for drug delivery may address the difficulty of reaching deep hypoxic regions (87).

#### 3.5.5 Neutrophils

Neutrophils, the predominant leukocytes in the circulatory system, are essential for acute inflammation and host defense against infections or tissue injury. They possess distinctive abilities to infiltrate inflamed brain tumors, which are not readily accessible to other cells. Neutrophils have demonstrated the ability to inhibit postoperative glioma recurrence by internalizing paclitaxel (PTX). Nonetheless, these nanoparticles may exhibit deficiencies, including diminished cell viability, inadequate cell quantities, and a potential risk of *in vitro* contamination. To enhance the therapeutic efficacy of neutrophil-based drug delivery systems (DDSs), researchers have suggested the *in situ* hitch-hiking of circulating neutrophils for cancer therapy. They encapsulated nanoparticles with bacteria-derived outer membrane vesicles to create neutrophil-mediated delivery systems containing nano-pathogenoids, capable of eradicating residual microtumors post-PTT. This strategy augmented the neutrophil count in tumors by 300–600% through PTT pretreatment, yielding a 60% tumor-free rate and a 97% tumor growth inhibition rate. Nanomedicines engineered for high binding affinity to neutrophils are essential for active targeting. Neutrophil membrane proteins, including anti-CD11b antibody-coated nanoparticles, can enhance *in vivo* phagocytosis by neutrophils. Researchers have devised a technique to target CD11b, a protein present on activated neutrophils, by encapsulating nanoparticles with anti-CD11b antibodies. This system, referred to as “Trojan horses,” engulfs neutrophils and enhances their uptake tenfold both *in vitro* and *in vivo*. This method, referred to as anti-CD11b-coated nanoparticles, is crucial for neutrophil adhesion and migration (88). This neutrophil-mediated drug delivery facilitates the transport of therapeutic agents to tumor metastases (TMEs) alongside neutrophil infiltration prompted by photosensitization. Nevertheless, the selective targeting of neutrophils using high-affinity ligands presents a challenge because of their close lineage similarity to other myeloid cells (89).

#### 3.5.6 EVs-derived NPs

Extracellular vesicles (EVs) are diminutive, membrane-enclosed vesicles that enable intercellular communication and the transport of cellular cargo. They exist in multiple bodily fluids and can be categorized into three subtypes: exosomes, microvesicles (exosomes), and apoptotic bodies. The dimensions of EVs differ, with exosomes (EXOs) measuring between 30 and 100 nm, microvesicles (MVs) ranging from 50 to 1,000 nm, and apoptotic bodies spanning from 50 nm to 5 μm. The characteristics and roles of a vesicle are contingent upon the cell type and the specific vesicle type. Exosomes originate from multivesicular bodies and are generated when the plasma membrane fuses with the endoplasmic reticulum. MVs or ectosomes arise from the rupture of the plasma membrane. Extracellular vesicles consist of proteins, lipids, and nucleic acids, predominantly RNA. They govern physiological processes, modulate the immune system, and can circumvent immune responses owing to their low immunogenicity and cytotoxicity. They can produce either anti-inflammatory or pro-inflammatory effects, contingent upon the cell type. When enveloped in EV membranes, nanoparticles can be obstructed by the immune system, facilitating improved targeting of the desired location. The nucleus of nanoparticles can be organic or inorganic, contingent upon the requisite properties and characteristics for a specific application. Inorganic cores comprise magnetic nanoparticles (MNPs), quantum dots (QDs), gold nanoparticles (AuNPs), and silica nanoparticles (SiNPs). MNPs consist of iron oxide or alternative magnetic substances and can be integrated into EVs or utilized independently for targeted delivery and hyperthermia therapy. Quantum dots possess distinctive optical characteristics, gold nanoparticles provide exceptional optical and plasmonic attributes, and silicon nanoparticles are favorable for biocompatibility (90). MSC-derived EVs (extracellular vesicles) are frequently utilized in wound healing owing to their capacity to regulate the immune response and facilitate tissue regeneration. These extracellular vesicles promote cell proliferation and angiogenesis, serving as facilitators of cellular communication. They have been examined for diverse applications in organs such as the skin, heart, kidneys, liver, and other tissues. EXOs, spheroidal entities encased in a lipid bilayer, are extensively utilized owing to their diagnostic and therapeutic capabilities, ease of manipulation, high stability, intrinsic capacity to target specific cells or tissues, multi-cargo loading potential, and diminutive size (30–200 nm). Exosomes are secreted by diverse cells and are present in biological fluids including breast milk, blood, urine, saliva, cerebrospinal fluid, and amniotic fluid. They possess drug-delivery capabilities, especially in immunotherapy for cancer treatment, and are suitable for gene delivery owing to their abundance of miRNA and mRNA. Recent studies indicate that stem cell-derived exosomes can enhance cell migration, proliferation, differentiation, re-epithelialization, and angiogenesis by activating particular signaling pathways. The utilization of exosomes or their membranes to encapsulate nanoparticles for regenerative medicine and tissue engineering is beneficial owing to their biological characteristics, such as low immunogenicity, biocompatibility, minimal toxicity, and the capacity for molecular exchange. Recent gels containing either exosomes or nanoparticles coated with exosome membranes have proven highly effective in wound healing. Studies have shown that MSC-derived exosomes exhibit surface markers CD9, CD63, and CD81, potentially facilitating wound healing in knockout mouse models. Nanoparticles coated with exosomal membranes provide various advantages, including extended circulation time in the bloodstream, enhanced biocompatibility, and superior targeted delivery (91). Exosome membranes emulate natural cell membranes, enabling nanoparticles to remain in the bloodstream for extended periods and access target tissues. They exhibit exceptional biocompatibility, thereby reducing the risks associated with immune responses. Exosome membranes possess distinct surface proteins and markers that can identify and attach to specific receptors on target cells, such as cancer cells. This precise delivery guarantees that the nanoparticles arrive at the designated cells, minimizing toxicity and off-target effects (92).

## 4 Unlocking novel pathways: mechanisms of action in precision oncology

### 4.1 Targeted drug delivery and tumor microenvironment modulation

The TME constitutes a significant obstacle to effective cancer therapy, comprising diverse cell subtypes, immune cells, endothelial and inflammatory cells, fibroblasts, lymphocytes, and chemokines. It plays a role in tumor initiation, metastasis, and recurrence. Immune cells are integral to both innate and adaptive immunity, with innate immunity facilitated by macrophages and dendritic cells. The TME is a multifaceted ecosystem that facilitates tumor cell proliferation, invasion, and metastasis, while also affecting drug resistance, immune evasion, and therapeutic efficacy. Distinct attributes such as hypoxia, acidic conditions, and elevated mesenchymal pressure can establish physical impediments to traditional drug delivery and treatment. Comprehending TME complexity is crucial for the development of effective targeted drug delivery systems (93) (Table 3).

**TABLE 3:** Innovative molecular pathways of nanoparticles for cancer treatment.

Nanoparticle type	Molecular mechanism	References
metal-based nanoparticles (like TiO2, ZnO, and silver),PLGA nanoparticles	Innate Immune System Activation: Nanoparticles, including titanium dioxide, zinc oxide, and zirconium dioxide, can engage with TLRs on immune cells, initiating the synthesis of inflammatory cytokines and other immune mediators. They can also initiate the complement system, provoking opsonization and the secretion of inflammatory mediators. Phagocytosis transpires when nanoparticles are internalized by phagocytic cells, which subsequently present antigens to the adaptive immune system. These cells secrete cytokines, including TNF-α, IL-1β, and IL-6, which are essential for inflammation and the recruitment of immune cells.	(94)
PLGA Iron oxide Gold and Liposomes nanoparticles	Adaptive Immune System Activation: Nanoparticles can stimulate the adaptive immune system by facilitating antigen presentation, thereby delivering antigens to antigen-presenting cells (APCs), such as dendritic cells. This stimulates T and B lymphocytes. Nanoparticles serve as vaccine delivery systems by encapsulating antigens and facilitating their controlled release, thereby enhancing the immune response. They can also be designed to target specific immune cells or tissues.	(95)
Gold nanoparticles, liposomes, and polymer-based nanoparticles	Targeting cancer stem cells AuNPs can be modified with targeting molecules, such as antibodies or peptides, to specifically bind to cancer stem cells (CSCs), including CD133, a marker for glioblastoma stem cells. They may also be integrated with photothermal therapy to augment their therapeutic efficacy. Liposomes, which are lipid-based nanoparticles, can encapsulate and deliver drugs to CSCs, with enhancements to increase their specificity. Polymeric nanoparticles, including chitosan, PLGA, and PEGylated polymers, are applicable for targeting cancer stem cells, with chitosan-based nanoparticles potentially targeting malignant cells while preserving healthy tissues. Inorganic nanoparticles, such as silica and iron oxide, are being investigated for targeting CSCs, with silica designed for effective drug delivery and iron oxide utilized in conjunction with magnetic hyperthermia to eliminate CSCs. Ultimately, stimuli-responsive nanoparticles, including redox-sensitive nanoparticles and temperature-sensitive liposomes, discharge their payload within the tumor microenvironment or directly within CSCs.	(96)

#### 4.1.1 Targeted delivery of nanocarriers in the hypoxic zone

Hypoxia, a prevalent characteristic of the TME, generates hypoxic zones within tumors, restricting cellular proliferation and enhancing resistance to radiotherapy and chemotherapy. Nanotechnology can resolve this issue by employing hypoxia-sensitive nanocarriers to enable targeted drug release or to detect diminished oxygen levels. Hypoxia-sensitive nanocarriers can be engineered to transport prodrugs and release active pharmaceuticals exclusively under hypoxic conditions, thereby reducing off-target effects and enhancing drug concentration. These nanoparticles can be designed to release drugs in reaction to particular chemical alterations in hypoxic conditions, such as elevated acidity or the presence of enzymes. Examples encompass fluorocarbon chain-functionalized hollow mesoporous organosilica nanoparticles (FHMONs), hemoglobin-based nanoprobes, pH-sensitive nanocarriers, and redox-responsive nanocarriers, which can mitigate hypoxia, augment photodynamic therapy, release drugs in the acidic milieu of tumors, and transport antioxidants or therapeutic agents. Superparamagnetic iron oxide nanoparticles can convey oxygen and release it in hypoxic areas, enhancing local oxygenation and the effectiveness of radiation therapy. Reduction-sensitive nanoparticles administer drugs via a reaction with glutathione, augmenting tumor cytotoxicity (97, 98).

#### 4.1.2 Smart nanocarriers for acidic microenvironments

The acidic environment of tumor cells, resulting from aberrant metabolism, influences their behavior and the effectiveness of chemotherapy. To tackle this issue, pH-responsive nanocarriers have been explored, encompassing polymer nanoparticles, liposomes, hydrogen-based systems, and metal-organic frameworks. pH-responsive nanocarriers encompass polymer nanoparticles, dendrimers, polymeric micelles, nanogels, liposomes, and pH-sensitive liposomes. DOPE: CHEMS liposomes, metal-organic frameworks (MOFs), covalent organic frameworks (COFs), and hybrid liposome/MOF systems. These nanocarriers can encapsulate pharmaceuticals and release them in response to pH fluctuations, with liposomes being spherical vesicles composed of lipid bilayers. Liposomes containing ionizable groups alter their charge and characteristics in reaction to pH, resulting in drug release. DOPE: CHEMS liposomes employ the CHEMS component, which undergoes protonation in acidic conditions, leading to liposome destabilization and subsequent drug release. Metal-organic frameworks (MOFs) are crystalline substances engineered for pH responsiveness, whereas covalent organic frameworks (COFs) possess a covalent architecture that can be synthesized to exhibit pH responsiveness. These nanocarriers can undergo depolymerization in acidic environments, facilitating drug release or targeted drug delivery. Certain nanoparticles may aggregate into larger entities, whereas others can regulate drug release. Hydrogels, HMSNs, MOFs, and charge-reversible nanoparticles represent promising drug delivery systems for tumors. Hydrogels can be engineered with pH-sensitive linkages that degrade in acidic environments, thereby releasing encapsulated pharmaceuticals. Chitosan-based hydrogels can be modified with pH-responsive polymers to facilitate controlled drug release in the acidic environment of tumors. HMSNs provide an extensive surface area for drug encapsulation and an adjustable pore size for regulated release. MOFs, which are porous crystalline materials, can be engineered with pH-sensitive linkers or specific pore dimensions to facilitate controlled drug release under acidic conditions. Charge-reversible nanoparticles can modify their surface charge in response to pH fluctuations, thereby improving cellular uptake in tumors via electrostatic interactions with the negatively charged cell membrane (99, 100).

#### 4.1.3 Nanosystems for high interstitial pressures

The TME is defined by the proliferation of tumor cells and their extracellular matrix, resulting in elevated interstitial pressure. This limits drug infiltration and obstructs the distribution of therapeutics and nanoparticles. Researchers have devised nanotechnology approaches to enhance drug delivery efficacy and target tumor microenvironment characteristics. These include modifying nanocarriers to enhance permeability, transporting enzymes that degrade the extracellular matrix, and engineering multifunctional nanocarriers. The amalgamation of immuno-oncology and nanomedicine has undergone evaluation in clinical trials, encompassing the “cancer-immunity” cycle (101).

### 4.2 Overcoming multidrug resistance in cancer cells

Tumor drug resistance poses a considerable challenge in cancer treatment, attributable to mechanisms including the overexpression of efflux proteins, enhanced DNA repair capabilities, and evasion of apoptosis. Nanotechnology has created sophisticated drug delivery systems to mol resistance gene expression, activation of the immune system, and facilitation of cell apoptosis. Nanocarriers are capable of encapsulating anticancer agents, including doxorubicin and paclitaxel, and can degrade under acidic or enzymatic conditions. They can also reverse chemotherapeutic drug resistance by administering inhibitors of ABC transporter proteins. Nanocarriers function as an ideal medium for the concurrent delivery of multiple drugs or therapeutic agents, enhancing the vulnerability of tumor cells to chemotherapy. These systems provide opportunities to augment immunotherapy and improve the therapeutic efficacy of tumor immunotherapy (102, 103).

### 4.3 Gene and RNA-based therapies using nanoparticles

RNA-based therapies are employed to address a range of medical conditions, encompassing mRNA therapy, cancer immunotherapy, and genetic disorders. These pharmaceuticals employ RNA interference mechanisms to modulate protein synthesis without altering the genome. RNA-based therapies encompass mRNA vaccines, siRNA therapies, and various other RNA molecules. LNPs are frequently utilized for RNA delivery, particularly in mRNA vaccines. Polymeric nanoparticles, composed of synthetic polymers, can encapsulate or conjugate with RNA molecules. AuNPs can be modified with RNA molecules for gene silencing or other therapeutic applications. Alternative nanomaterials such as silica, iron oxide, and dendrimers may also serve as carriers for RNA delivery. CRISPR-based therapies entail the administration of plasmids that harbor the CRISPR-Cas9 system or mRNA that encodes the Cas9 protein and guide RNA (sgRNA). Nanoparticles can facilitate gene activation and silencing by delivering small activating RNA (saRNA) to activate specific genes or small interfering RNA (siRNA) to inhibit them (104). Recent research indicates that acidic buffers can improve the stability of lipid nanoparticles, rendering them appropriate for mRNA delivery. Liu et al. formulated a charge-assisted stabilization strategy for lipid nanoparticles, while Han’s team created AID-lipids for mRNA delivery (105). LNPs show potential for the delivery of antisense oligonucleotides (ASOs) but encounter obstacles in attaining significant mRNA downregulation because of ASO accumulation in the ventricles and blood vessels. LNPs may elicit an immune response owing to their distinctive lipid composition, which can engage with immune cells and induce inflammation. Researchers are investigating alternative nanoparticle systems, including polymer-based nanoparticles, inorganic nanoparticles, hybrid nanoparticles, and peptide nano-assemblies, to overcome these limitations. These systems provide adjustable characteristics, regulated release, and potential for precise delivery and imaging applications. The objective is to reduce toxicity while enhancing delivery efficiency, taking into account variables such as particle size, surface charge, and interactions with biological systems (106). These carriers provide advantages such as extrahepatic delivery, chemical diversity, and design versatility. Future investigations should concentrate on developing more efficient RNA delivery mechanisms and incorporating peptide nano-assemblies (107). Nanoparticle delivery systems, such as lipid nanoparticles, polyion complex micelles, pH-responsive polymer nanoparticles, and biodegradable polymer nanoparticles, facilitate the transport of nucleic acid therapeutics. These systems safeguard nucleic acids from degradation and facilitate cellular trafficking. LNPs are crucial for mRNA vaccines, safeguarding mRNA from degradation and enhancing cellular uptake. Nevertheless, merely approximately 2% of LNPs discharge their cargo into the cytosol, constraining their effectiveness. The low efficiency of cytosolic delivery is essential for optimizing the effectiveness of these delivery systems. Instances of lipid nanoparticles utilized in COVID-19 vaccines comprise ALC-0315 and SM-102. Research continues to enhance lipid nanoparticle formulations for more effective and targeted delivery of mRNA and other nucleic acids (108).

### 4.4 PIC micelles

PIC micelles are delivery systems utilizing block copolymers, consisting of a neutral hydrophilic block and a cationic block, capable of responding to alterations in the biological environment such as pH, redox conditions, temperature, and salt concentration (109).

### 4.5 pH-responsive polymer nanoparticles

pH-responsive nanoparticles regulate nucleic acid delivery by altering pH levels resulting from endocytosis. When combined with a block copolymer, they self-assemble into nanoparticles ranging from 80 to 200 nm. Engineered for oligonucleotide vaccination and DNA delivery, these biodegradable polymer nanoparticles can encapsulate nucleic acids, yet may provoke immune responses and inflammation (110).

### 4.6 Biodegradable polymeric nanoparticles

Biodegradable polymer nanoparticles, including Poly (d, l-lactide-co-glycolic acid) (PLGA), can encapsulate nucleic acids during the synthesis of the particles. PLGA is an FDA-approved synthetic biodegradable polymer utilized in diverse biomedical applications, including drug delivery systems such as nucleic acid delivery. Its biodegradability and biocompatibility render it optimal for encapsulation during synthesis. PLGA nanoparticles can encapsulate and deliver diverse nucleic acids, including siRNA, DNA, and Peptide Nucleic Acids (PNAs), for gene editing and the targeting of specific genes. PLGA undergoes hydrolysis within the body, decomposing into lactic acid and glycollic acid monomers, thereby releasing the nucleic acid payload. These nanoparticles have been effectively loaded with nucleic acid therapies, facilitating controlled release and prolonged gene expression. Nonetheless, PLGA polymers present difficulties in modification and may elicit inflammation and immune reactions (111).

### 4.7 Immunomodulation and cancer vaccination with nanocarriers

NPs have garnered attention as delivery vehicles for cancer vaccines owing to their capacity to encapsulate or affix antigens to surfaces, thereby ensuring a sustained immune response. The design of therapeutic cancer vaccines is enhanced by diverse nanomaterials, such as lipid-based, protein-based, polymer-based, inorganic-based, and bio-inspired carriers. Lipid-based nanoparticles, such as cationic liposomes, have demonstrated safety and efficacy in human immunotherapies and animal tumor models. Protein-based nanoparticles are advantageous for the development of subcellular vector proteins owing to their biodistribution and biocompatibility. Polymeric nanoparticles, including polymeric micro/nanoparticles, micelles, dendrimers, nanodiscs, and hydrogels, have been thoroughly investigated for vaccine delivery. Synthetic polymeric nanoparticles exhibit enhanced immune responses and are utilized in gene delivery (112). Mesoporous silica nanoparticles (MSNPs) and bio-inspired delivery vehicles (OMVs) exhibit potential in oncological therapy owing to their robust immunostimulatory characteristics and stability. MSNPs possess a porous architecture, elevated surface area, and efficient loading capacity, whereas OMVs can display various antigens and modulate the tumor microenvironment without adverse effects. Ghost vaccines provide secure administration, adaptability, and maintenance of antigenicity, whereas OMVs focus on antigens, small interfering RNAs, peptide antigens, and nanoparticles. Further research is essential to ascertain safety, biocompatibility, and to prolong vaccine half-life (113).

### 4.8 Photothermal and photodynamic therapy: next-generation strategies

**FIGURE 2:**
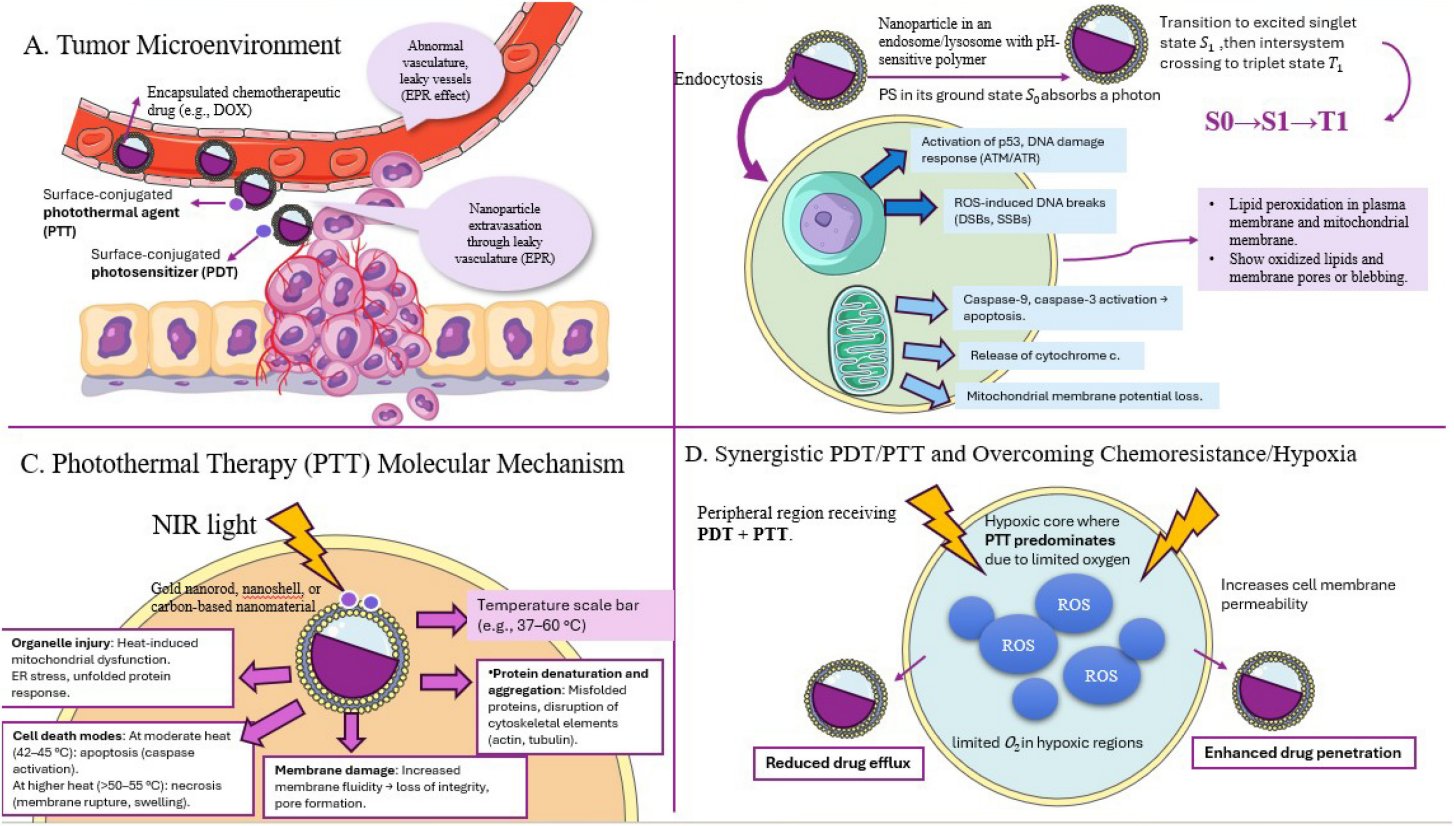
Molecular mechanisms of nanoparticle-mediated photodynamic and photothermal cancer therapy. **(A)** Tumor targeting and nanoparticle accumulation. Multifunctional nanoparticles carrying a photosensitizer (PS), photothermal agent, and chemotherapeutic payload accumulate in tumor tissue through the enhanced permeability and retention (EPR) effect and ligand–receptor mediated targeting. The nanoparticles enter cancer cells primarily via endocytosis within the heterogeneous tumor microenvironment characterized by abnormal vasculature, hypoxia, and acidic pH. **(B)** Photodynamic therapy (PDT) mechanism. Upon light irradiation, the photosensitizer transitions from the ground state (S_0_) to an excited singlet state (S_1_) and subsequently to a triplet state (T_1_) via intersystem crossing. The excited triplet state transfers energy or electrons to molecular oxygen through type II or type I photochemical reactions, producing reactive oxygen species (ROS) such as singlet oxygen (^1^O_2_), superoxide (●O_2_), and hydroxyl radicals (●OH). These ROS induce lipid peroxidation, mitochondrial membrane damage, cytochrome-c release, caspase activation, and DNA damage, ultimately triggering apoptosis. **(C)** Photothermal therapy (PTT) mechanism. Photothermal agents absorb near-infrared (NIR) light and convert photon energy into localized heat through non-radiative relaxation processes. The resulting hyperthermia disrupts cellular homeostasis by inducing protein denaturation, membrane destabilization, mitochondrial dysfunction, and cytoskeletal damage. Depending on temperature intensity, these effects lead to apoptosis or necrosis of tumor cells. **(D)** Synergistic therapeutic effects. The combined PDT/PTT strategy enhances anticancer efficacy by integrating ROS-mediated oxidative damage with heat-induced cellular stress. Photothermal heating can increase membrane permeability, improve drug penetration, and partially overcome hypoxia-associated limitations of PDT. Together, these processes promote efficient tumor cell killing while enabling spatially controlled activation at the irradiated tumor site.

Phototherapies are cancer treatments whereby photochemical or photothermal changes within a target tissue are induced by light. Two often used phototherapies produce cytotoxic ROS or local temperature increase: photodynamic (PDT) and photothermal (PTT) therapy. Compared to radiation therapy, PDT and PTT can complement mainstay cancer treatments, overcome chemotherapy resistance, modify tumor perfusion, and vascular and extracellular matrix permeability, improve tumor drug delivery, provide greater spatiotemporal control, and lower the risk of secondary cancer development (Figure 2). PDT utilizes oxygen, a light-activated agent, and light to produce cytotoxic ROS that initiate biological processes. Photodynamic therapy (PDT) is appropriate for tumors located near critical structures, as it minimally impacts nerves and the extracellular matrix. PDT and photothermal therapy are two clinically significant photodynamic therapies. PDT exhibits greater selectivity owing to its dual selectivity for photosensitizer accumulation and light targeting, whereas PTT focusses on infiltrative and non-resectable tumor components. Photodynamic therapy (PDT) is predominantly oxygen-independent, rendering it appropriate for the treatment of hypoxic tumors. The efficient delivery of photoactive agents can be achieved by incorporating organic dyes into nanostructures. Porphysomes, liposomal nanoparticles, and human serum albumin-ICG conjugates exemplify dual photodynamic therapy/photothermal therapy agents utilizing a singular photoactive agent (114). The stability of porphyrin quenching in lipoprotein nanoparticles is contingent upon the nature of the lipid conjugates, with amphiphilic lipids exhibiting reduced quenching and enhanced ROS generation. Regulating the photophysical properties of nanoparticles is difficult. To balance these characteristics, Zhao and colleagues (115) developed a discretely integrated nanofabrication (DIN) platform combining cisplatin, ICG, and a polymeric spacer (PES). The utilization of dual PDT/PTT nanoparticles, integrating inorganic materials with various photosensitizing agents, has enabled the simultaneous application of chemotherapy, PTT, and PDT. Innovative methods are being devised to regulate the properties of nanoparticle PDT/PTT, achieving effective treatment of 4T1 tumors with polypyrrole-tellurophene nanoparticles. Mesoporous silica nanoparticles provide avenues for the integration of therapeutic agents, while multifunctional phototheranostic agents facilitate the simultaneous application of PDT and PTT (116).

## 5 Personalized and precision nanomedicine for cancer

### 5.1 Tailoring nanoparticles for individual patient profiles

Precision medicine, a concept associated with personalized medicine, was established in 2015 by the US National Research Council and President Obama’s Precision Medicine Initiative. It enables physicians to forecast disease progression and adjust preventative strategies, potentially reducing the occurrence or advancement of tumors. Precision medicine encompasses disease management and prevention as well. Optimizing a drug’s pharmacokinetics and biodistribution enhances its delivery to target cells or disease sites while minimizing off-target effects. Precision oncology focusses on individualized treatment strategies that integrate clinical data, molecular attributes, and genetic profiles. The MOSAIC project seeks to create an extensive database of spatial omics data in oncology, elucidating cancer subtypes, drug targets, and biomarkers. Notwithstanding recent FDA approvals, challenges persist in the clinical implementation of these medications, including immunological toxicities and inadequate targeting efficacy. Contemporary technologies are being devised to enhance the precision and efficacy of these therapies. Dual PDT/PTT nanoparticles utilizing a singular photoactive agent can be synthesized, with more advanced methodologies currently in progress. Multifunctional phototheranostic agents can streamline the concurrent use of PDT and PTT, yet attaining optimal relative tumor absorption remains challenging (114, 117). Table 4 illustrates the clinical application of some NPs in cancer therapy.

**TABLE 4:** Clinical application of main NPs in cancer therapy.

Nanoparticle type	Clinical application	References
Gold nanoparticles (AuNPs)	AuNPs facilitate targeted drug delivery, improve permeability and retention, and enable photothermal therapy by selectively adhering to cancer cells, passively accumulating in tumor tissues, and converting near-infrared light into heat, ultimately leading to the destruction of cancer cells. They serve as contrast agents in imaging modalities such as computed tomography (CT) and photoacoustic imaging, markedly improving tumor identification.	(118)
Liposomes	Liposomes, spherical vesicles composed of lipid bilayers, can encapsulate pharmaceuticals and transport them to tumor locations. They can be designed to deliver drugs at a regulated rate, enhancing drug efficacy and minimizing side effects, exemplified by Doxil, an FDA-approved medication for ovarian cancer and Kaposi’s sarcoma.	(119)
Magnetic Nanoparticles (MNPs)	MNPs are applicable in hyperthermia therapy, drug delivery, and imaging. They can be heated with an alternating magnetic field to eradicate cancer cells and serve as contrast agents for MRI.	(120)
carbon nanotubes (CNTs)	Carbon nanotubes can be modified for drug delivery, targeted therapy, imaging, and combination therapy. They can be functionalized with targeting agents such as antibodies or folic acid to selectively bind to cancer cells, thereby enhancing treatment efficacy. They may also serve as contrast agents for imaging modalities such as MRI, facilitating enhanced tumor detection and monitoring. CNTs can be integrated with additional therapies such as photothermal or photodynamic therapy. Examples encompass folate-functionalized CNTs, magnetic CNTs for targeted therapy and MRI contrast enhancement, and CNT-based siRNA delivery to inhibit specific genes implicated in cancer progression.	(121)
Quantum dots (QD)	Quantum dots (QDs) are semiconductor nanocrystals characterized by distinctive optical properties, rendering them advantageous for imaging and diagnostics. They provide enhanced sensitivity, multiplexed imaging, and intricate visualization of biological processes. QDs exhibit size-tunable emission, elevated brightness, distinct absorption spectra, surface functionalization capabilities, and quantum confinement effects. They are utilized in *in vitro* and *in vivo* imaging, early disease detection, tumor targeting, drug administration, and theranostics.	(122)
Chitosan nanoparticles (CSNPs)	CSNPs serve as biocompatible and biodegradable carriers for targeted drug delivery across diverse biomedical applications. They can be engineered to target specific cells or tissues, enable controlled release, and exhibit versatility. They are especially beneficial for tumor treatment, gene therapy, wound healing, and vaccine administration. CSNPs have been studied for the treatment of conditions such as cancer, diabetes, and cardiovascular diseases. Challenges encompass scalability, regulatory frameworks, targeting efficiency, and drug loading capacity. Future directions encompass the establishment of regulatory frameworks, enhancement of targeted delivery precision, and emphasis on drug loading capacity.	(123)
Calcium phosphate nanoparticles	Calcium phosphate nanoparticles exhibit biocompatibility and biodegradability, rendering them suitable for drug delivery applications. Their resemblance to bone and teeth facilitates secure integration into the body for precise drug delivery within cells and tissues. These nanoparticles can efficiently transport therapeutic agents, including nucleic acids, proteins, and pharmaceuticals, to designated locations within the body. Their solubility, which is dependent on pH, facilitates controlled drug release. They can be synthesized in diverse sizes and morphologies, and their surface can be modified for improved drug loading and targeting. Applications encompass cancer treatment, bone regeneration, gene delivery, and dental and orthopedic implants owing to their biocompatibility.	(124)
Albumin-bound nanoparticles (Abraxane)	Abraxane is a chemotherapeutic agent comprising albumin-bound nanoparticles of paclitaxel, utilized for the treatment of multiple cancers. This formulation is specifically designed to optimize the delivery of paclitaxel to cancer cells, potentially increasing its efficacy and minimizing adverse effects. Abraxane’s nanoparticle formulation utilizes the body’s inherent albumin transport systems to enhance drug delivery to tumor cells. It provides numerous benefits compared to conventional paclitaxel formulations, such as superior solubility, optimized delivery, heightened efficacy, and diminished toxicity. Abraxane obviates the necessity for solvents, enhancing its safety for administration. Clinical trials indicate that Abraxane may exhibit greater efficacy in specific cancer types and potentially result in reduced toxicity relative to solvent-based paclitaxel.	(125)

### 5.2 Role of artificial intelligence in designing smart nanocarriers

Emerging as interesting substitutes for traditional delivery systems, smart delivery systems (SMNs) solve problems including non-specific distribution and uncontrollably released cargo. Because these systems are stimuli-responsive, therapeutic agents can be delivered effectively and controlled release at the target site is enabled. To completely realize SMNs’ potential, though, more study is required. Transforming many disciplines, including biomedicine, artificial intelligence (AI) lets machines and computers replicate human cognitive processes. By simulating biological processes at several phases—target identification, SMN, payload design, biodistribution, and simulation of extracellular and intracellular interactions—AI helps to design and optimize SMNs. Essential in this process are Deep Learning (DL) and Machine Learning (ML), which help to predict interactions between nanocarriers and biological systems and analyze challenging data patterns. Combining several treatments under one smart system by AI assistants results in more efficient patient care and tailored medicine. Innovative modeling platforms and systems resulting from the integration of artificial intelligence into SMN manufacture maximize material selection, predict performance, and generate more efficient and customized drug and gene distribution systems. Targeting efficiency, biodistribution, and possible toxicity of SMNs, AI models can help save time and money by predicting these factors. *In vivo* studies can thus be avoided. Polymers for effective gene-editing payload delivery have been identified using combinational design and ML; artificial intelligence-driven image analysis methods have been developed to measure intracellular delivery of therapeutic agents by nanocarriers. Theranoscopic multifunctional nanocarriers combine therapeutic and diagnostic roles into one nanocarrier. By optimizing their dual functionality, which enables personalized treatment by changing drug dosages, targeting mechanisms, and diagnostic parameters depending on the patient’s unique biological profile, artificial intelligence greatly increases the theranostic performance. Real-time patient response to treatment by artificial intelligence algorithms also allows dynamic changes to treatment plans. Therapeutic agent delivery is much hampered by biological barriers including the tumor microenvironment and the BBB. Employing optimal design parameters, behavior of nanocarriers in complex biological environments, and identification of strategies to improve their penetration and distribution, artificial intelligence can help to overcome these constraints. By foretelling the density and orientation of target ligands on the nanocarrier surface, artificial intelligence models maximize binding to target cells and effective intracellular trafficking. AI can maximize nanocarriers in tumor tissues and reduce off-target effects in cancer treatment by optimizing their accumulation. Furthermore, improving molecular profiling and enabling early cancer case detection is artificial intelligence. Challenges including regulatory approval, scalability, and cost-effectiveness define the clinical translation of AI-driven SMNs (126, 127).

### 5.3 Theranostic nanoparticles: dual functionality for diagnosis and therapy

Theranostics, which integrate therapeutic and diagnostic methodologies, are anticipated to transform personalized medicine by facilitating concurrent monitoring and treatment of breast cancer at cellular or molecular levels. The integration of ultrasound and MRI characteristics can produce more precise information. Photothermal therapy, employing near-infrared lasers and photo absorbers, is garnering attention as an alternative to traditional treatments owing to its minimal invasiveness and superior controllability. Theranostic nanoparticles, comprising polymer-based nanostructures and gold nanostructures, exhibit promise for cancer theranostics owing to their biocompatibility and distinctive optical and electrical properties (128). NPs are categorized according to their size, morphology, composition, and physicochemical properties. Inorganic nanoparticles, including metal oxides (MO), metal-organic frameworks (MOFs), gold nanoparticles (AuNPs), lanthanide-doped nanoparticles (LnNPs), and silicon nanoparticles (SiNPs), exhibit potential in cancer therapy by inducing cytotoxicity in cancer cells via reactive oxygen species generation and photothermal effects. Iron oxide nanoparticles are widely utilized in imaging and therapy owing to their unique properties. Wang et al. engineered superparamagnetic iron oxide nanoparticles (SPIO NPs) for targeted therapy in lung cancer cells, demonstrating that functionalized nanoparticles accumulate substantially in tumor tissues, thereby enhancing therapeutic efficacy and reducing energy consumption. Iridium oxide nanoparticles have been utilized for *in vivo* fluorescence imaging and integrated chemo- and photothermal therapy. Zhang et al. investigated iridium oxide nanoparticles for *in vivo* fluorescence imaging and integrated chemotherapy with photothermal therapy. Metal-organic frameworks (MOFs) are a category of organic-inorganic hybrid porous materials characterized by customizable dimensions, elevated porosity, diverse compositions and morphologies, substantial surface areas, and significant porosity. Gold nanoparticles are photothermally active entities utilized in cancer imaging and therapy, as well as potential drug carriers, owing to their high atomic number, radiation-enhancing properties, ease of size regulation, and surface functionalization capabilities. Lanthanide-doped nanoparticles exhibit excellent optical and thermal stability, along with tunable luminescence emission, rendering them appropriate for photodynamic therapy, drug delivery, and bioimaging applications. Zhou et al. (129) developed Lipo-FNPs by loading a carboxyl-containing ferrocene derivative on the surface of UCLNPs (NaYF4:Yb, Tm@NaYF4:Eu) and encapsulating them into liposomes. A nano-platform integrating liposomes and lanthanide-doped materials was evaluated for upconversion luminescence imaging and near-infrared-promoted photo-Fenton therapy of hypoxic tumors. The system’s therapeutic efficacy arises from the overexpression of H2O2 in the tumor microenvironment, facilitating exceptional specificity and minimal harm to healthy tissues. Li et al. created a high-performance theranostic nanoplatform utilizing NRs@PDA for near-infrared II tumor-associated blood vessel imaging and photothermal therapy ablation, demonstrating substantial tumor growth inhibition. The combined administration of chemo- and photothermal components is a promising approach to reduce the time of treatment and concentration of DOX (130, 131).

Ferreira et al. (132) developed ultrasmall silicon-based nanoparticles to effectively exploit the EPR effect. Ultrasmall porous silica nanoparticles (UPSN) were covered with DOTA, a chelator that binds the radionuclides 90 Y and 86Y for radiotherapy and PET imaging. The *in vivo* therapeutic effect of 90Y-DOTA-UPSN showed a time-progressive accumulation in tumor mass with a maximum tumor/muscle ratio at 48 h. Wang et al. (133) reported a nano platform composed of PLGA NPs covered with manganese dioxide (MnO_2_) ultrathin nanofilms for MRI and X-ray computed tomography (CT), loaded with gold nanorods (AuNRs) for radio frequency (RF) hyperthermia, and with docetaxel (DTX) for chemotherapy. The final nano construct (PLGA/AuNR/DTX@MnO_2_) was assessed for theranostic applications in a breast cancer cell line, xenograft mice model, *in vitro* toxicity evaluation, and dual-modal imaging. The combination of AuNRs-induced RF hyperthermia and DTX-induced chemotherapy could reach efficient tumor inhibition. Li et al. (134) documented the application of PLGA-based nanoparticles for photoacoustic imaging (PAI) and photothermal therapy (PTT), utilizing a copolymeric polyethylene glycol-PLGA nanosystem infused with near-infrared cocaine. The nanoparticles were coated with iRGD peptide for targeted delivery. The photothermal conversion efficiency was determined to be 4.0 times greater at pH 5.8 than at pH 7.4. The cytotoxicity of PLGA-NPs was assessed *in vitro* using breast cancer cells. The nanoparticles exhibited an effective photothermal therapeutic effect. Dong et al. synthesized PLGA-based nanoparticles encapsulating perfluorooctyl bromide (PFOB) and superparamagnetic iron oxide particles, coated with a gold nanoshell. Her2-GPH nanoparticles were utilized for dual-modal imaging via ultrasound/MRI and photothermal therapy. The Her-2 dependent active targeting was validated *in vitro* in Her-2 positive breast cancer cells (SKBR3) in contrast to Her-2 negative cell lines. The Her2-GPH nanoparticles exhibited a high signal-to-noise ratio and contrast in both imaging modalities. Notwithstanding the encouraging *in vitro* outcomes for dual-modal imaging via US/MRI, *in vivo* investigations are requisite to comprehensively ascertain the potential of these nanoparticles in oncological therapy. Chitosan, a natural pH-sensitive polymer, is utilized in diverse applications owing to its biocompatibility, biodegradability, and elevated cellular uptake. It can be readily transformed into nanoparticles, creating a stable polyplex with both chemotherapeutic and therapeutic agents in a singular process.

Gholami et al. (135) created chitosan-based nanoplatforms utilizing a poly-L-arginine-chitosan-triphosphate matrix (ACSD) as a vehicle for doxorubicin and superparamagnetic iron oxide nanoparticles (SPIONs). These nanoparticles exhibited pH-dependent release of DOX for targeted cancer therapy and demonstrated MRI properties. Biological nanoparticles, including liposomes, consist of biological macromolecules and membranes derived from viral capsids or red blood cells. Liposomes received FDA approval in 1995 as a nanomedicine platform for the treatment of AIDS-related Kaposi’s sarcoma. Feng et al. (136) created a liposome-based apparatus containing a hypoxia-activated prodrug (AQ4N) and a hydrophobic hexadecyl amine-conjugated chlorin e6 (hCe6), which serves as a photosensitizer. The substantial loading capacity of liposomes was utilized to encapsulate a significant quantity of both the AQ4N prodrug and the 64Cu-hCe6 photosensitizer. The liposome-based device, a nanoplatform, has demonstrated potential in improving cellular uptake and photodynamic therapeutic efficacy in breast cancer cells. The nanoplatform, composed of graphene oxide flakes reinforced liposomes (GOF-Lipo) conjugated with folic acid (FA) and encapsulated with doxorubicin (DOX-GOF-Lipo-FA), was evaluated for photothermal and chemothermal therapy, resulting in a significant suppression of tumor growth *in vivo*. The fabrication of nanoparticles employs proteins such as BSA and HAS. Zhao et al. (137) created an albumin-based photothermal therapy nanoplatform by conjugating human serum albumin (HSA) with a P-selectin-targeting peptide (PSN peptide) and IR780. The research investigates the application of virus-like particles (VLPs) and red blood cell membranes (RBCs) in diverse fields such as cancer therapies, immunotherapies, vaccines, cardiovascular procedures, gene therapies, imaging, and theranostics. The researchers employed the Tobacco Mosaic Virus (TMV) to fabricate serum albumin-coated nanoparticles (NPs) for theranostic purposes, demonstrating notable biodistribution in breast cancer cell lines and *in vivo* within a heterotopic breast cancer model. The research also created RBC-derived magnetic carriers to improve drug delivery and imaging, and investigated optical nanostructures from erythrocytes infused with the near-infrared dye indocyanine green (NETs) for near-infrared fluorescence imaging and photodestruction (138, 139).

## 6 Preclinical and clinical advances in next-generation nanoparticle therapies

### 6.1 *In vitro* and *in vivo* studies on advanced nanoparticles

Liposomes serve as efficient drug delivery systems owing to their capacity to encapsulate hydrophobic, hydrophilic, and amphiphilic pharmaceuticals. They are non-toxic, hypoallergenic, non-antigenic, and biodegradable. Recent studies have developed pH-sensitive liposomes conjugated with cyclic arginine-glycine-aspartate (cRGD) and encapsulated with doxorubicin, demonstrating significant antitumor efficacy. Micelles, amphiphilic molecules characterized by a hydrophilic exterior and hydrophobic interior, have been utilized in numerous cancer therapies. Protein-based nanoparticles, including Abraxane, Pegaspargase, gelatin nanoparticles, silk fibroin, and sorafenib, have demonstrated significant effectiveness in targeting cancer cells. Polymeric nanoparticles, such as dendrimers, polyamidoamine (PAMAM) dendrimers, chitosan, and synthetic polymeric nanoparticles, have demonstrated significant effectiveness in targeting cancer cells. Immune cells, including dendritic cells, macrophages, and T lymphocytes, can recognize and eradicate cancer cells via their engagement with the immune system. Macrophages and their derivatives have been examined as carriers for cancer therapeutics (140). CAR-T therapy and dendritic cell-based vaccines demonstrate promise in personalized medicine for patients with acute lymphocytic leukemia. Infiltrated T-cells can be isolated from tumors and cultured as active tumor targets, yielding autologous antitumor lymphocytes. DC-based vaccines, such as PROVENGE, are genetically modified to present tumor antigens and augment antitumor immune responses. Viruses, including viral nanoparticles and virus-like particles, can provoke immune responses due to their antigenic properties. These technologies hold potential for targeted cancer therapies; however, they encounter challenges concerning immunogenicity, stability, and drug delivery limitations. Bacteria have been utilized as drug delivery systems for cancer therapies, leading to tumor reduction (141, 142). A scientist has utilized products derived from bacteria to treat almost 1,000 patients, including Coley’s Toxins, which stimulate the immune system and facilitate tumor elimination via immunotherapy. The immunotherapy agent axalimogene filolisbac (AXAL) was created by attenuating a strain of Listeria monocytogenes to specifically target neoplastic cells infected with HPV. Bacterial ghosts (BGs) are empty bacterial shells obtained from Gram-negative bacteria such as E. coli, providing safety, cost-efficiency, and immunostimulatory characteristics. A recent study demonstrated that BGs serve as carriers for the delivery of anti-cancer drugs targeting non-Hodgkin’s lymphoma cells. Minicells and SimCells serve as drug delivery systems for cancer treatment. Minicells are engineered to synthesize azurin, a peptide possessing anti-cancer properties, while SimCells are genetically altered to produce I-CeuI (143–145).

Bacterial outer membrane vesicles (OMVs) are spherical structures released by gram-negative bacteria during their exponential growth phase. They comprise proteins, RNA, DNA, and lipopolysaccharides. OMVs possess immunogenic properties and can specifically target and accumulate in tumor tissues, rendering them advantageous. They have exhibited antitumor effects across various cancer types, as evidenced by *in vivo* murine models and *in vitro* investigations. OMVs have been utilized as drug delivery systems, demonstrating substantial tumor suppression, self-blockade, and strong anti-tumoral efficacy. Macrophage-encapsulated OMVs have been utilized to deliver Ce6 and doxorubicin to the 4T1 breast cancer cell line (146). Table 5 illustrates preclinical and clinical advancements in nanoparticle therapies.

**TABLE 5:** Preclinical and clinical advances nanoparticle therapies.

Nanoparticle type	Clinical advances	Nanoparticle type	Preclinical advances	References
Liposomal doxorubicin (Myocet)	Approved for the treatment of metastatic breast carcinoma.	Thermosensitive liposomes (TSLs)	Lyso-thermosensitive liposomal doxorubicin (LTLD) facilitates improved drug delivery to tumors.	(147, 148)
Albumin-bound paclitaxel (Abraxane)	Approved for metastatic breast carcinoma, non-small cell lung carcinoma, and pancreatic carcinoma	Lipid-based Nanoparticles for mRNA Delivery	These demonstrate potential in cancer immunotherapy by administering mRNA that encodes tumor antigens.	(149, 150)
Liposomal Vincristine (Marqibo)	Approved for Philadelphia chromosome-negative acute lymphoblastic leukemia.	Functionalized Liposomes	Liposomes can be altered with antibodies and cell-penetrating agents to enhance drug delivery.	(151, 152)
Liposomal Daunorubicin (DaunoXome)	Approved for Kaposi’s sarcoma	Iron Oxide Nanoparticles	These are utilized in preclinical studies for targeted tumor imaging, drug delivery, and hyperthermia therapy.	(153, 154)
Genexol-PM	A polymeric micelle formulation of paclitaxel has been approved for the treatment of non-small cell lung cancer, metastatic breast cancer, and ovarian cancer.			(155)

### 6.2 FDA-approved nanoparticle therapies

Table 6 enumerates the Nanopharmaceuticals approved by the U.S. The FDA and EMA utilize 56% of lipid-based nanoformulations for oncological therapies, with liposomes being the predominant choice owing to their versatility, biocompatibility, and non-immunogenic characteristics. Conventional liposomes exhibit restricted blood circulation and preferentially accumulate in the liver and spleen, thereby hindering their ability to target tumor tissue. Numerous nanopharmaceuticals, such as Doxil*™*, Caelyx*™*, and Myocet*™*, employ lipid-based nanotechnology platforms. Nano pharmaceuticals have been engineered to improve the safety profile of drugs such as Doxorubicin and extend effective tumor therapy. These nanoparticles offer advantages including biocompatibility, biodegradability, and cost-effectiveness. Protein-based nanoparticles can convey genetic materials, anticancer drugs, peptide hormones, growth factors, DNA, and RNA. Oncaspar*™* is a PEGylated formulation of the enzyme asparaginase that degrades and reduces the concentration of the amino acid asparagine, which is essential for tumor cell proliferation. Ontak*™* is a recombinant fusion toxin originating from diphtheria toxin, which obtained FDA approval in 1999 for the treatment of human CD25 + cutaneous T cell lymphoma (CTCL). Eligard*™* is a gonadotropin-releasing hormone (GnRH) agonist utilized for advanced hormone-dependent prostate cancer, breast cancer, and various other conditions. Abraxane*™* and Pazenir*™* are albumin-bound formulations of Paclitaxel that elicit cytotoxicity by inhibiting mitosis through microtubule stabilization, resulting in cellular apoptosis. Kadcyla*™* is the first antibody-drug conjugate (ADC) approved by the FDA and EMA in 2013. Additional ADCs comprise polatuzumab vedotin-piiq, Brentuximab vedotin, tisotumab vedotin-tftv, gemtuzumab ozogamicin, Enfortumab vedotin, Sacituzumab govitecan, Trastuzumab deruxtecan, loncastuximab tesirine-lpyl, Moxetumomab pasudotox, belantamab mafodotin-blmf, and inotuzumab ozogamicin. Nanopharmaceuticals such as Lipusu™, Genexol™, and Nanoxel™ are marketed regionally and indicated for multiple cancer types. NanoThermTM is the exclusive metallic-based cancer therapy that has obtained FDA and EMA approvals. Magnetic nanoparticles are directly administered into the tumor or resection cavity wall, obliterating cancer cells or enhancing their sensitivity for radiotherapy or chemotherapy to prevent recurrences. Nanomedicine is continuously evolving, with nearly 30,000 articles published since the early 2000s and new nanopharmaceuticals undergoing clinical investigation annually (156–158)

**TABLE 6:** Catalog of FDA-approved nanodrugs for cancer treatment.

Nano drugs	Brand name	Company	Substance	Application	Year(s) of approval
Irinotecan	Onivyde^®^	Merrimack	Liposome	Pancreatic cancer	2015
Leuprolide acetate	Eligard^®^	Tolmar	PLGA	Prostate cancer	2002
Doxorubicin	Doxil^®^	Janssen	Liposome-PEG	Metastatic breast and ovarian cancers	1995
Paclitaxel	Genexol PM^®^	Samyang Corporation	mPEG-PLA	Metastatic breast cancer	2007
Paclitaxel	Abraxane^®^	Celgene	Albumin	Metastatic breast cancer	2005
Doxorubicin	Caelyx™	Johnson and Johnson	Liposome-PEG	Advanced epithelial ovarian cancer	2004
Myocet	Myocet™	Jiwan	Non-PEGylated, liposomal	Metastatic breast cancer	1999

### 6.3 Case studies of successful nanoparticle-based cancer treatments

Nanotechnology concentrates on the development of materials for drug delivery systems aimed at tumor treatment, utilizing diverse substances such as polymeric nanoparticles, liposomes, micelles, hydrogels, exosomes, and others. Hybrid materials, including gold nanoparticles, amalgamate the advantages of diverse materials. Natural polymeric nanocarriers such as chitosan and human serum albumin are frequently employed in the architecture of nanomedicine. Synthetic polymers such as polylactic acid (PLA) and poly (lactic-co-glycolic acid) (PLGA) are widely employed in tumor therapy owing to their prolonged circulating half-life and sustained release properties. Dendrimers possess a three-dimensional, repetitively branched architecture that demonstrates remarkable hydrophilicity, non-toxicity, bioavailability, drug stability, enhanced biological activities, and superior transmembrane efficacy. Additional trials are required to overcome limitations associated with tumor mesenchymal stem cells (TMS) and to improve drug delivery methods (159). Liposomes provide precise drug delivery, reduce systemic toxicity, and ensure stable drug environments. Research indicates that conjugation with EphA2 antibodies can diminish the toxicity of antitumor agents, enhance tolerability, and augment efficacy. Micelles are spherical structures formed by self-aggregating amphiphilic molecules exhibiting specific properties. The proliferation of micelle-based pharmaceuticals indicates a promising future in clinical applications (160). Hydrogels are biocompatible, versatile polymer chains employed in multiple medical fields, such as tissue engineering, wound healing, and cancer immunotherapy. They replicate healthy tissues and can be produced from natural or synthetic polymers. Nanohydrogels provide benefits such as biodegradability and renal elimination. They also hold promise for anticancer pharmaceutical applications. EVs, membrane-bound particles, are biologically important for cellular functions and intercellular communication, with prospective applications in cancer diagnosis and therapy (161). Tumor-derived extracellular vesicles (TEVs) and immune cell-derived extracellular vesicles are promising candidates in tumor immunotherapy owing to their immunogenic characteristics. These vesicles can provoke an immune response against neoplastic cells, activate immune cells, and suppress immune responses. Natural membrane-coated NPs are under examination for drug delivery, with RBC NPs employed to enhance blood circulation and optimize targeting of cancer cells. Leukocyte-mimetic carriers and platelet membrane-coated nanoparticles are under investigation for cancer therapies (162). Plant virus-like particles (VLPs) and inorganic nanoparticles exhibit promise in immunotherapy and vaccination. Inorganic nanoparticles possess distinct physical, chemical, and biological properties, such as controlled release, biocompatibility, and stability. Mesoporous silica nanoparticles (MSNs) offer benefits such as chemical modification, anticancer drug encapsulation, and an uncomplicated manufacturing process. MSNs can associate with siRNA and PEI, thereby suppressing tumor gene expression. Gold nanoparticles, due to their optical properties and Surface Plasmon Resonance characteristics, have significant applications in cancer diagnosis and treatment (163). Gold nanoparticles, generated via synthesis methods, possess diverse properties and applications, such as photothermal therapy, photodynamic therapy, and photo imaging. They exhibit biocompatibility, are non-toxic, and possess no adverse effects. Carbon nanomaterials, such as carbon dots and graphene, possess a wide range of applications in biomedical carriers, composites, electronics, and sporting goods. The covalent functionalization of carbon nanotubes has been employed to create stimulus-responsive capping techniques. Tailored materials-based nanotechnology is meeting tumor therapy requirements by comprehending tumor pathological traits and microenvironments (164). Nanotechnology has augmented the efficacy of traditional chemotherapy by improving drug retention, targeting neoplastic sites, and enhancing specificity. PEGylated lipid nanoparticles have demonstrated a tenfold enhancement in platinum circulation within the bloodstream. Nanocarriers can promote synergistic chemotherapy, reduce drug resistance, improve pharmacokinetics, and augment anti-tumor efficacy. Albumin-bound paclitaxel and gemcitabine are endorsed regimens for the treatment of pancreatic cancer. An innovative FH-SSL-Nav liposome specifically targets the tumor matrix, facilitating infiltration, reducing ECM deposition, and enhancing blood perfusion (165). Together, Table 7 summarizes the important case studies related to nanotechnology against cancers.

**TABLE 7:** Case studies in nanotechnology for cancer therapy.

Nanotechnological platform	Key findings and advantages	Cancer-related application	Reference(s)
Tumor mesenchymal stem cell (TMS)-based nanocarriers	Require further trials to overcome delivery limitations; potential for improved tumor-specific drug transport	Experimental tumor-targeted drug delivery	(158)
Liposomes (incl. antibody-conjugated)	Precise drug delivery, reduced systemic toxicity, improved drug stability; EphA2-antibody conjugation enhances tolerability and efficacy	Targeted delivery of antitumor agents	(159)
Polymeric micelles	Self-assembled amphiphilic nanostructures; growing number of micelle-based pharmaceuticals; strong clinical translation potential	Drug solubilization, delivery of hydrophobic anticancer drugs	(159)
Hydrogels/nanohydrogels	Biocompatible, mimic tissue environment; suitable for tissue engineering, wound healing, immunotherapy; nanohydrogels show biodegradability and renal elimination	Delivery of anticancer agents, cancer immunotherapy	(160)
Natural membrane-coated nanoparticles (RBC-NPs, leukocyte-mimetic, platelet-coated)	Enhance circulation time; improve targeting; biomimetic immune-evasive characteristics	Targeted drug delivery, tumor microenvironment modulation	(161)
Plant virus-like particles (VLPs)	Strong immunogenicity, useful in vaccine development	Cancer immunotherapy and vaccination	(162)
Inorganic nanoparticles	Unique physical/chemical features; controlled release, high biocompatibility, stability	General anticancer drug delivery, imaging	(162)
Mesoporous silica nanoparticles (MSNs)	Easy functionalization; efficient drug encapsulation; siRNA and PEI loading suppresses tumor gene expression	Gene therapy, chemotherapy, siRNA delivery	(162)
Gold nanoparticles (AuNPs)	Surface plasmon resonance; used in PTT, PDT, imaging; highly biocompatible and non-toxic	Photothermal therapy, photodynamic therapy, cancer imaging	(162)
Carbon nanomaterials (carbon dots, graphene, CNTs)	High versatility; functionalizable for stimuli-responsive delivery	Drug delivery, smart nanocarriers, responsive therapeutic systems	(163)
PEGylated lipid nanoparticles	10 × increase in platinum circulation; improved pharmacokinetics and tumor targeting	Enhanced chemotherapy delivery (e.g., platinum drugs)	(163)
FH-SSL-Nav liposome	ECM-targeting properties; improves infiltration, reduces matrix deposition, enhances perfusion	Enhanced penetration of tumors with dense stroma (e.g., pancreatic)	(164)
Albumin-bound paclitaxel + gemcitabine	Clinically endorsed combination regimen; better tumor accumulation via albumin transport pathways	Pancreatic cancer therapy	(164)

## 7 Challenges and future directions in next-generation cancer nano therapy

### 7.1 Scalability and large-scale manufacturing of nanoparticles

**FIGURE 3:**
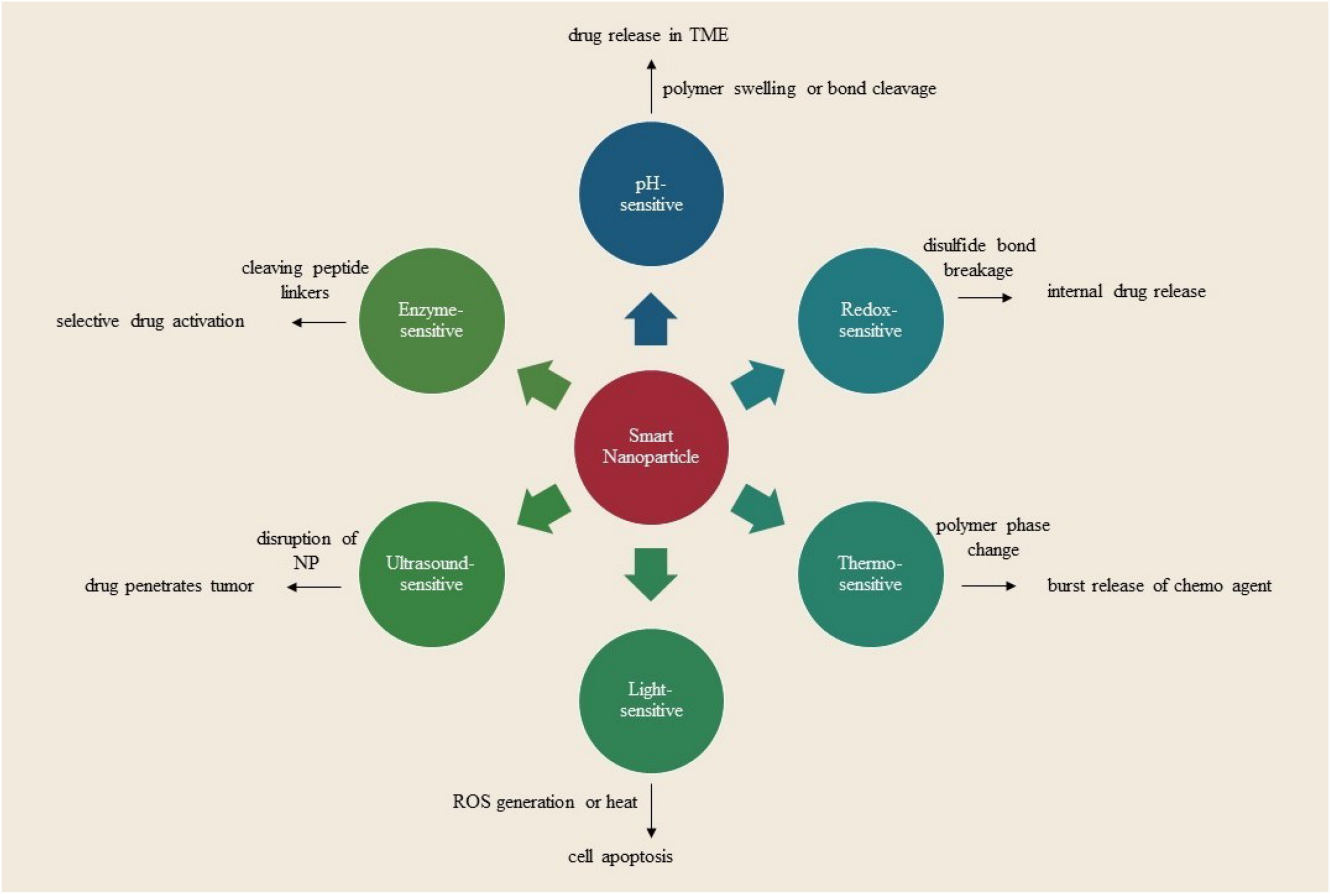
Overview of smart nanocarriers designed to respond to tumor-specific stimuli for on-demand drug release. Various external and internal cues, including acidic pH, high glutathione concentrations, enzymatic activity, elevated temperature, light, and ultrasound, trigger structural changes or cleavage reactions in the nanocarriers, leading to controlled and localized release of therapeutic agents within the tumor microenvironment. These systems aim to improve therapeutic precision while minimizing off-target toxicity.

Nanomedicine products can be transitioned from laboratory to market through bottom-up and top-down methodologies (Figure 3). The bottom-up approach is less favored because of the challenges of eliminating solvent residues. Factors including material composition, toxicological characteristics, *in vivo* biodegradability, and the equilibrium of multicomponent systems are essential. Meticulous selection of materials, solvents, and production techniques is crucial for optimizing time efficiency and achieving the desired characteristics of nanoparticles. Upon optimizing therapeutic requirements, market demand, research, development, production processes, scalability, clinical trials, and regulatory considerations, nanomedicine products are introduced to the market. Nanomedicine products available in the market include Abraxane^®^, Estrasorb, Triglide, Doxil^®^, Ambisome^®^, Emend, Daunoxome, and MegaceES. Nanoparticle-based formulations for *in vivo* imaging comprise Gastromark, RTricor, esovist, and Feridex. Table 8 depicts all reported obstacles to the clinical application of nanoparticles for cancer treatment (39, 166).

**TABLE 8:** Obstacles in the clinical application of nanoparticles for cancer treatment.

Obstacles	References
Challenges in securing regulatory approval for the market release of the drug	(167)
Inadequate residence duration within the organism	(168)
Inability to extrapolate *in vitro* findings to *in vivo* investigations	(169)
Inadequate loading of the drug within the nanoparticles	(170)
The protracted procedure of pharmaceutical development	(171)
The inability of the drug formulation to preferentially concentrate at the target site	(172)
Concerns regarding biocompatibility and toxicity	(173)
Obstacles in cellular absorption	(174)
Deficiencies in loading, internalization, and drug release	(175)
Duration necessary for preclinical and clinical research involving higher animals and humans	(176)
Formulation instability	(177)
Insufficient biodegradation and removal	(178)

### 7.2 Safety, toxicity, and biocompatibility concerns

NMs intended for biomedical applications require assessment for cytotoxicity and biocompatibility, necessitating a thorough evaluation both *in vitro* and *in vivo*. These tests are essential to guarantee the safety of patients undergoing NM-based therapies and manufacturing personnel. Comprehending the correlations between the physicochemical characteristics of nanomaterials and their distinct biological effects is crucial for expanding their applications. Nanomaterials have demonstrated therapeutic applications, including the regulation of scarring, mitigation of fibrosis, and inhibition of cancer proliferation. Nevertheless, comprehending the bio-interactions of nanomaterials is complex due to the diverse array of material types, biological systems, and analytical metrics that measure their interactions. Cytotoxicity assays evaluate compromised cell membranes, generating cell-death indicators, and disrupted metabolic processes. Examples encompass the LDH assay for ZnO nanoparticles, the Caspase-3/7 assay for caspases, and the MTT assay for cellular viability. Nanomaterials can diminish cell viability by causing DNA damage and disrupting metabolic functions, contingent upon their composition or geometry. CuO nanoparticles have been documented as cytotoxic, inflicting greater damage to DNA and mitochondria, resulting in cell death. Nanomaterial geometry also affects cytotoxicity through ROS generation. A material that does not influence cell mortality may still elicit sub-lethal effects on the genome and epigenome, particularly at low doses. Comprehending the bio-interactions of nanomaterials is crucial for expanding their applications and mitigating adverse effects. NMs have been investigated for their genotoxic effects, immunomodulatory properties, and potential to induce fibrosis. Nano cellulose, derived from the degradation of larger cellulose structures, exhibits a diverse array of molecular weights and dimensions. The molecular weights can vary from several hundred to several thousand Daltons (g/mol) for oligomers, and exceed 500,000 Da for native cellulose. Factors affecting molecular weight encompass the type of plant or source material, the preparation technique, and the reaction conditions. Various forms of Nano cellulose (CNC, CNF, BC) possess distinct inherent molecular weight properties. Molecular weight determination can be achieved through techniques such as Size Exclusion Chromatography (SEC), Matrix-Assisted Laser Desorption/Ionization Time-of-Flight Mass Spectrometry (MALDI-TOF MS), and Ultracentrifugation. Alternative techniques encompass the end-group method, osmotic pressure method, and viscosity method. The molecular weight distribution of Nano cellulose is affected by the type of plant or source material, the preparation method, and the reaction conditions (179). Conventional methodologies for evaluating nanomaterials encompass the Ames test, comet assay, micronucleus assay, DNA laddering assay, and chromosomal aberration assay. Nanomaterials can induce genomic alterations and facilitate intracellular generation of hydroxyl radicals, resulting in increased reactive oxygen species and DNA damage. Examples of nanomaterials that elevate ROS levels include silver, titanium dioxide, silica, quantum dots, and carbon nanotubes. Surface charges influence the genotoxicity of liposomes, with positively charged nanocarriers exhibiting reduced genotoxicity. NMs can result in both direct and indirect immunomodulation, encompassing immunosuppression and immunostimulation. This position is especially critical in drug delivery applications, where nanoparticles function as carriers for vaccines or pharmaceuticals. The immune response elicited by nanoparticles encompasses both innate and adaptive immunity. The intricate process affected by factors including chemical composition, molecular structure, surface chemistry, and size has been examined. The size of nanoparticles is pertinent to this process; as larger particles provoke more robust immune responses. Bulk material possessing spiky nanostructures has demonstrated the ability to modulate the immune response. Fibrosis, characterized by the excessive accumulation and restructuring of dense ECM, may arise in response to NMs. NMs can provoke fibrosis via multiple mechanisms, including the elevation of ROS, inflammation, and heightened expression of TGF-β. The therapeutic application of nanoparticles has emerged from their capacity to penetrate biological membranes, and their cytotoxic properties have been utilized to address conditions related to excessive cellular proliferation (180, 181).

### 7.3 Regulatory and ethical considerations for nanomedicine in oncology

The ethical principles in nanomedicine, including respect for autonomy, beneficence, no maleficence, and justice, are compromised. Beauchamp and Childress’ bioethical theory asserts a dialectical relationship between ethical principles and specific ethical dilemmas, resulting in a revised perspective on real ethical issues. These principles, universally recognized across cultures, are deemed *prima facie* binding, signifying they must be adhered to unless they conflict with an equally compelling or superior principle. The authors contend that no principle is superior to the others, and the prioritization of principles is contingent upon the specific context of the situation. They have created an “ethical matrix” to facilitate the application of their theory to practical inquiries. The majority of ethical inquiries on nanomedicine have been addressed by the principles established by Beauchamp and Childress. An illustration of employing their principles as instruments for scrutinizing ethical dilemmas in nanomedicine may involve an ethical evaluation of utilizing nanoparticles for early disease diagnosis and the administration of therapeutic agents. In this scenario, the principles of no maleficence, beneficence, and respect for autonomy are in opposition, necessitating that agents deliberately ascertain which principles should be disregarded in the specific context. The theory of Beauchamp and Childress, significant in bioethics, is open to philosophical scrutiny. Hedgecoe critiques conventional philosophical bioethics for emphasizing idealized rationality while neglecting social and cultural influences. He promotes “critical bioethics,” in which empirical research contests and subverts the theoretical framework. Nonetheless, the criticism lacks a solid basis. Beauchamp and Childress contend that a dialectical relationship exists between ethical principles and ethical dilemmas, whereby the emergence of new ethical issues may necessitate a critical examination and redefinition of ethical principles. The principles of Beauchamp and Childress are adaptable rather than fixed, necessitating supplementation with paradigm cases, empirical evidence, and organizational experience. Rights, virtues, and emotional responses are crucial for ethical judgment. The indeterminate nature of Beauchamp and Childress’ theory renders it appropriate for addressing nascent ethical dilemmas in nanomedicine. The theory’s responsiveness to the dynamics of nanotechnology enables it to effectively tackle emerging ethical concerns (182).

### 7.4 Future innovations: smart, self-assembling, and biodegradable nanoparticles

Intelligent NPs present a viable alternative to conventional nanoparticles in cancer treatment, as they can be activated by specific stimuli and directed to precise locations for targeted drug delivery. These nanoparticles aggregate at the targeted location and release their payloads, establishing an intelligent therapeutic mechanism. They can concurrently administer therapeutics and diagnostic agents, promoting the progression of theranostics and sophisticated nanoparticles for cancer therapy. Diverse nanocarriers exhibit unique structures and attributes, facilitating the selection of suitable nanocarriers according to the drug’s properties and therapeutic needs. Nanocarriers such as liposomes, micelles, polymeric nanoparticles, and solid lipid nanoparticles possess distinctive characteristics that influence drug loading, release, and targeting efficacy. The selection is contingent upon the drug’s characteristics and the intended therapeutic result. Researchers can employ these principles to develop sophisticated and efficient drug delivery systems for improving targeted therapies. Bioavailability profoundly influences the effectiveness of anticancer treatments, and sophisticated drug delivery systems, such as drug nanocarriers, have been developed to enhance the therapeutic potential of encapsulated chemotherapeutic agents by mitigating undesirable properties and improving pharmacokinetics and tissue distribution. Biodegradable nanoparticles, including PLGA, are extensively utilized for the targeted delivery of pharmaceuticals, vaccines, and various biomolecules. Active targeting of nanoparticles is crucial for precise drug delivery to specific targets, with molecular recognition mechanisms such as ligand-receptor specificity and antigen-antibody interactions being fundamental. Oral drug delivery utilizing nanoparticles exhibits superior bioavailability, extended residence time, and enhanced biodistribution compared to free drugs (183–185).

## 8 Benefits of engineered nanoparticles in food preservation

Beyond biomedical applications especially in cancer treatment, the increasing demand for safe, high-quality, and minimally processed foods has stimulated the development of innovative preservation technologies. Among these, engineered nanoparticles have emerged as promising tools for improving food stability, safety, and shelf-life (186). Nanotechnology enables the design of nanoscale materials with unique physicochemical properties, including high surface area, tunable surface chemistry, and enhanced reactivity. These features allow nanoparticles to interact more efficiently with microbial cells, food components, and environmental factors that contribute to food spoilage. Consequently, engineered nanoparticles are increasingly explored for their capacity to enhance food preservation strategies while maintaining nutritional quality and sensory attributes (187). One of the most important advantages of engineered nanoparticles in food preservation is their ability to act as efficient carriers for bioactive compounds such as antioxidants, antimicrobial agents, vitamins, and essential oils (188). Many natural preservatives suffer from poor stability, low solubility, or rapid degradation during food processing and storage. Encapsulation of these compounds within nanoparticles can significantly improve their stability against environmental stressors such as light, oxygen, temperature, and pH variations (189). Furthermore, nanoencapsulation enables controlled and sustained release of active compounds, ensuring prolonged antimicrobial or antioxidant activity and thereby reducing the rate of food spoilage (190).

Engineered nanoparticles also exhibit strong antimicrobial properties, which play a critical role in preventing microbial contamination and extending food shelf-life (191). Certain nanomaterials, including metal and metal oxide nanoparticles as well as biopolymer-based nanostructures, can disrupt microbial cell membranes, generate reactive oxygen species, or interfere with cellular metabolic processes (192). These mechanisms can inhibit the growth of foodborne pathogens and spoilage microorganisms, thereby enhancing food safety. When incorporated into food packaging materials or edible coatings, antimicrobial nanoparticles can create active packaging systems that continuously protect food products from microbial contamination during storage and distribution.

Another important application of engineered nanoparticles is in the development of advanced food packaging systems (193). Nanotechnology allows the fabrication of nanocomposite materials with improved mechanical strength, gas barrier properties, and thermal stability compared with conventional packaging materials. By reducing the permeability of oxygen, moisture, and other gases, nanoparticle-reinforced packaging can effectively slow oxidative reactions and microbial growth, preserving the freshness and quality of food products (193). Additionally, nanoparticles can be incorporated into intelligent packaging systems capable of sensing environmental changes, detecting microbial contamination, or indicating food spoilage, thereby providing real-time monitoring of food quality (194). Biopolymer-based nanoparticles, particularly those derived from natural polymers such as chitosan, alginate, cellulose, and starch, have attracted significant attention due to their biocompatibility, biodegradability, and environmental sustainability (195). These materials offer a safe and eco-friendly alternative to synthetic preservatives and conventional packaging technologies (196). Engineered biopolymer nanoparticles can be tailored to improve adhesion to food surfaces, enhance encapsulation efficiency of bioactive compounds, and regulate release kinetics (11). As consumer preference increasingly shifts toward natural and clean-label food products, the integration of engineered nanoparticles, especially biodegradable biopolymer nanostructures, represents a promising and sustainable strategy for modern food preservation.

## 9 Conclusion and future directions

Notwithstanding progress in cancer treatment, there remains a necessity for more efficacious therapies. Nanomedicines exhibit significant potential in oncology, yet considerable opportunities remain to be explored. Progress in formulation, surface functionalization, and comprehension of the biological behavior of nanomaterials *in vivo* will facilitate the introduction of additional formulations into clinical trials for cancer therapy. Additional investigation into large-scale production and stability will augment the success of Nanomedicine in oncology. Nanoparticle drug delivery systems provide substantial benefits compared to free drugs by regulating *in vivo* drug disposition, enhancing drug efficacy, and minimizing off-target side effects. Progress in targeting strategies has resulted in the creation of novel formulations, emphasizing surface functionalization through targeting ligands. Although 12 Nanomedicine therapies have received clinical approval for oncology, they predominantly emphasize entrapment and PEGylating to enhance active pharmaceutical ingredient performance. Beyond biomedical and oncological applications, engineered biopolymer nanoparticles also offer substantial advantages in the field of food preservation. Their intrinsic biocompatibility, biodegradability, and environmental safety make carbohydrate- and polysaccharide-based nanostructures, such as chitosan, alginate, cellulose derivatives, and starch, attractive candidates for sustainable food protection systems. At the nanoscale, these materials can encapsulate and stabilize sensitive bioactive compounds, antimicrobials, antioxidants, and natural preservatives, shielding them from oxidation, heat, and photodegradation. Moreover, engineered biopolymer nanoparticles provide controlled and sustained release profiles, enabling prolonged antimicrobial activity, delaying spoilage, and extending shelf life without compromising sensory qualities. Their tunable surface chemistry further allows improved adhesion to food matrices and the creation of active packaging systems capable of inhibiting pathogenic growth and maintaining product freshness. As global demand intensifies for minimally processed foods and clean-label technologies, the engineering of biopolymer nanoparticles represents a promising, safe, and eco-friendly approach to modern food preservation. The integration of entrapment with active targeting and surface modifications of Nanomedicines may yield significant patient advantages, supported by enhanced manufacturing scalability and commercial feasibility.

## References

[1] Bray F Laversanne M Sung H Ferlay J Siegel R Soerjomataram I et al. Global cancer statistics 2022: globocan estimates of incidence and mortality worldwide for 36 cancers in 185 countries. *CA Cancer J Clin.* (2024) 74:229–63. 10.3322/caac.21834 38572751

[2] Siegel R Kratzer T Giaquinto A Sung H Jemal A. Cancer statistics, 2025. *CA Cancer J Clin.* (2025) 75:10–45. 10.3322/caac.21871 39817679 PMC11745215

[3] Tracey S Smyth P Barelle C Scott C. Development of next generation nanomedicine-based approaches for the treatment of cancer: we’ve barely scratched the surface. *Biochem Soc Trans.* (2021) 49:2253–69. 10.1042/BST20210343 34709394 PMC8589425

[4] Herdiana Y Wathoni N Shamsuddin S Muchtaridi M. Scale-up polymeric-based nanoparticles drug delivery systems: development and challenges. *OpenNano.* (2022) 7:100048. 10.1016/j.onano.2022.100048

[5] Din F Aman W Ullah I Qureshi O Mustapha O Shafique S et al. Effective use of nanocarriers as drug delivery systems for the treatment of selected tumors. *Int J Nanomedicine.* (2017) 12:7291–309. 10.2147/IJN.S146315 29042776 PMC5634382

[6] Ghazal H Waqar A Yaseen F Shahid M Sultana M Tariq M et al. Role of nanoparticles in enhancing chemotherapy efficacy for cancer treatment. *Next Mater.* (2024) 2:100128. 10.1016/j.nxmate.2024.100128

[7] Barenholz Y. Doxil^®^–the first FDA-approved nano-drug: lessons learned. *J Control Release.* (2012) 160:117–34. 10.1016/j.jconrel.2012.03.020 22484195

[8] Cheng H Liao J Ma Y Sarwar M Yang H. Advances in targeted therapy for tumor with nanocarriers: a review. *Mater Today Bio.* (2025) 31:101583. 10.1016/j.mtbio.2025.101583 40061211 PMC11889621

[9] Manikandan V Min S. Roles of polysaccharides-based nanomaterials in food preservation and extension of shelf-life of food products: a review. *Int J Biol Macromol.* (2023) 252:126381. 10.1016/j.ijbiomac.2023.126381 37595723

[10] Joye I McClements D. Biopolymer-based delivery systems: challenges and opportunities. *Curr Top Med Chem.* (2016) 16:1026–39. 10.2174/1568026615666150825143130 26303423

[11] Luo Y Wang Q Zhang Y. Biopolymer-based nanotechnology approaches to deliver bioactive compounds for food applications: a perspective on the past, present, and future. *J Agric Food Chem.* (2020) 68:12993–3000. 10.1021/acs.jafc.0c00277 32134655

[12] Cushen M Kerry J Morris M Cruz-Romero M Cummins E. Nanotechnologies in the food industry–recent developments, risks and regulation. *Trends Food Sci Technol.* (2012) 24:30–46. 10.1016/j.tifs.2011.10.006

[13] Khezerlou A Tavassoli M Alizadeh Sani M Mohammadi K Ehsani A McClements D. Application of nanotechnology to improve the performance of biodegradable biopolymer-based packaging materials. *Polymers.* (2021) 13:4399. 10.3390/polym13244399 34960949 PMC8707388

[14] Karahmet Sher E Alebić M Marković Boras M Boškailo E Karahmet Farhat E Karahmet A et al. Nanotechnology in medicine revolutionizing drug delivery for cancer and viral infection treatments. *Int J Pharm.* (2024) 660:124345. 10.1016/j.ijpharm.2024.124345 38885775

[15] Wu J. The enhanced permeability and retention (EPR) effect: the significance of the concept and methods to enhance its application. *J Pers Med.* (2021) 11:771. 10.3390/jpm11080771 34442415 PMC8402171

[16] Chen Z Kankala R Long L Xie S Chen A Zou L. Current understanding of passive and active targeting nanomedicines to enhance tumor accumulation. *Coord Chem Rev.* (2023) 481:215051. 10.1016/j.ccr.2023.215051

[17] Hou X Zaks T Langer R Dong Y. Lipid nanoparticles for mRNA delivery. *Nat Rev Mater.* (2021) 6:1078–94. 10.1038/s41578-021-00358-0 34394960 PMC8353930

[18] Mitchell M Billingsley M Haley R Wechsler M Peppas N Langer R. Engineering precision nanoparticles for drug delivery. *Nat Rev Drug Discov.* (2021) 20:101–24. 10.1038/s41573-020-0090-8 33277608 PMC7717100

[19] Danhier F Ansorena E Silva J Coco R Le Breton A Préat V. PLGA-based nanoparticles: an overview of biomedical applications. *J Control Release.* (2012) 161:505–22. 10.1016/j.jconrel.2012.01.043 22353619

[20] Dykman L Khlebtsov N. Gold nanoparticles in biomedical applications: recent advances and perspectives. *Chem Soc Rev.* (2012) 41:2256–82. 10.1039/c1cs15166e 22130549

[21] Xie J Lee S Chen X. Nanoparticle-based theranostic agents. *Adv Drug Deliv Rev.* (2010) 62:1064–79. 10.1016/j.addr.2010.07.009 20691229 PMC2988080

[22] Blanco E Shen H Ferrari M. Principles of nanoparticle design for overcoming biological barriers to drug delivery. *Nat Biotechnol.* (2015) 33:941–51. 10.1038/nbt.3330 26348965 PMC4978509

[23] Mura S Nicolas J Couvreur P. Stimuli-responsive nanocarriers for drug delivery. *Nat Mater.* (2013) 12:991–1003. 10.1038/nmat3776 24150417

[24] Fang R Kroll A Gao W Zhang L. Cell membrane coating nanotechnology. *Adv Mater.* (2018) 30:e1706759. 10.1002/adma.201706759 29582476 PMC5984176

[25] Monopoli M Aberg C Salvati A Dawson K. Biomolecular coronas provide the biological identity of nanosized materials. *Nat Nanotechnol.* (2012) 7:779–86. 10.1038/nnano.2012.207 23212421

[26] Cedervall T Lynch I Lindman S Berggård T Thulin E Nilsson H et al. Understanding the nanoparticle-protein corona using methods to quantify exchange rates and affinities of proteins for nanoparticles. *Proc Natl Acad Sci U S A.* (2007) 104:2050–5. 10.1073/pnas.0608582104 17267609 PMC1892985

[27] Prajapati A Rangra S Patil R Desai N Jyothi V Salave S et al. Receptor-targeted nanomedicine for cancer therapy. *Receptors.* (2024) 3:323–61. 10.3390/receptors3030016

[28] Argenziano M Arpicco S Brusa P Cavalli R Chirio D Dosio F et al. Developing actively targeted nanoparticles to fight cancer: focus on italian research. *Pharmaceutics.* (2021) 13:1538. 10.3390/pharmaceutics13101538 34683830 PMC8540327

[29] Singh R Srinivas S Kumawat M Daima H. Ligand-based surface engineering of nanomaterials: trends, challenges, and biomedical perspectives. *OpenNano.* (2024) 15:100194. 10.1016/j.onano.2023.100194

[30] Schwartz A. Receptor cell biology: receptor-mediated endocytosis. *Pediatr Res.* (1995) 38:835–43. 10.1203/00006450-199512000-00003 8618782

[31] Kumar A Ahmad A Vyawahare A Khan R. Membrane trafficking and subcellular drug targeting pathways. *Front Pharmacol.* (2020) 11:629. 10.3389/fphar.2020.00629 32536862 PMC7267071

[32] de Roode K Joosten L Behe M. Towards the magic radioactive bullet: improving targeted radionuclide therapy by reducing the renal retention of radioligands. *Pharmaceuticals.* (2024) 17:256. 10.3390/ph17020256 38399470 PMC10892921

[33] Riccardi F Dal Bo M Macor P Toffoli G. A comprehensive overview on antibody-drug conjugates: from the conceptualization to cancer therapy. *Front Pharmacol.* (2023) 14:1274088. 10.3389/fphar.2023.1274088 37790810 PMC10544916

[34] Ntellas P Athauda A Sugiyama K Le M Crespi V Chau I. Expanding the potential of antibody-drug conjugates in gastrointestinal malignancies: beyond HER2 targets. *ESMO Gastrointest Oncol.* (2025) 8:100154. 10.1016/j.esmogo.2025.100154 41646266 PMC12836551

[35] Kang S Kim S. Toxicities and management strategies of emerging antibody-drug conjugates in breast cancer. *Ther Adv Med Oncol.* (2025) 17:17588359251324889. 10.1177/17588359251324889 40151551 PMC11946287

[36] Shi J Kantoff P Wooster R Farokhzad O. Cancer nanomedicine: progress, challenges and opportunities. *Nat Rev Cancer.* (2017) 17:20–37. 10.1038/nrc.2016.108 27834398 PMC5575742

[37] Danhier F Feron O Préat V. To exploit the tumor microenvironment: passive and active tumor targeting of nanocarriers for anti-cancer drug delivery. *J Control Release.* (2010) 148:135–46. 10.1016/j.jconrel.2010.08.027 20797419

[38] Desgrosellier J Cheresh D. Integrins in cancer: biological implications and therapeutic opportunities. *Nat Rev Cancer.* (2010) 10:9–22. 10.1038/nrc2748 20029421 PMC4383089

[39] Mehta M Bui T Yang X Aksoy Y Goldys E Deng W. Lipid-based nanoparticles for drug/gene delivery: an overview of the production techniques and difficulties encountered in their industrial development. *ACS Mater Au.* (2023) 3:600–19. 10.1021/acsmaterialsau.3c00032 38089666 PMC10636777

[40] Nsairat H Khater D Sayed U Odeh F Al Bawab A Alshaer W. Liposomes: structure, composition, types, and clinical applications. *Heliyon.* (2022) 8:e09394. 10.1016/j.heliyon.2022.e09394 35600452 PMC9118483

[41] Tayeb H Felimban R Almaghrabi S Hasaballah N. Nanoemulsions: formulation, characterization, biological fate, and potential role against COVID-19 and other viral outbreaks. *Colloid Interface Sci Commun.* (2021) 45:100533. 10.1016/j.colcom.2021.100533 34692429 PMC8526445

[42] Ashfaq R Rasul A Asghar S Kovács A Berkó S Budai-Szûcs M. Lipid nanoparticles: an effective tool to improve the bioavailability of nutraceuticals. *Int J Mol Sci.* (2023) 24:15764. 10.3390/ijms242115764 37958750 PMC10648376

[43] Rahman M Jalouli M Bhajan S Al-Zharani M Harrath AHA. Comprehensive review of nanoparticle-based drug delivery for modulating PI3K/AKT/mTOR-Mediated autophagy in cancer. *Int J Mol Sci.* (2025) 26:1868. 10.3390/ijms26051868 40076496 PMC11899884

[44] Geszke-Moritz M Moritz M. Biodegradable polymeric nanoparticle-based drug delivery systems: comprehensive overview, perspectives and challenges. *Polymers.* (2024) 16:2536. 10.3390/polym16172536 39274168 PMC11397980

[45] Plucinski A Lyu Z Schmidt B. Polysaccharide nanoparticles: from fabrication to applications. *J Mater Chem B.* (2021) 9:7030–62. 10.1039/d1tb00628b 33928990

[46] Abourehab M Rajendran R Singh A Pramanik S Shrivastav P Ansari M et al. Alginate as a promising biopolymer in drug delivery and wound healing: a review of the state-of-the-art. *Int J Mol Sci.* (2022) 23:9035. 10.3390/ijms23169035 36012297 PMC9409034

[47] Ewii U Attama A Olorunsola E Onugwu A Nwakpa F Anyiam C et al. Nanoparticles for drug delivery: insight into in vitro and in vivo drug release from nanomedicines. *Nano TransMed.* (2025) 4:100083. 10.1016/j.ntm.2025.100083

[48] Hong S Choi D Kim H Park C Lee W Park H. Protein-based nanoparticles as drug delivery systems. *Pharmaceutics.* (2020) 12:604. 10.3390/pharmaceutics12070604 32610448 PMC7407889

[49] Nafari A Cheraghipour K Sepahvand M Shahrokhi G Gabal E Mahmoudvand H. Nanoparticles: new agents toward treatment of leishmaniasis. *Parasite Epidemiol Control.* (2020) 10:e00156. 10.1016/j.parepi.2020.e00156 32566773 PMC7298521

[50] Unnikrishnan G Joy A Megha M Kolanthai E Senthilkumar M. Exploration of inorganic nanoparticles for revolutionary drug delivery applications: a critical review. *Discov Nano.* (2023) 18:157. 10.1186/s11671-023-03943-0 38112849 PMC10730791

[51] Attia N El-Monaem E El-Aqapa H Elashery S Eltaweil A El Kady M et al. Iron oxide nanoparticles and their pharmaceutical applications. *Appl Surf Sci Adv.* (2022) 11:100284. 10.1016/j.apsadv.2022.100284

[52] Cotta M. *Quantum Dots and Their Applications: What Lies Ahead?.* Washington, DC: ACS Publications (2020). p. 4920–4.

[53] Eker F Akdaşçi E Duman H Bechelany M Karav S. Gold nanoparticles in nanomedicine: unique properties and therapeutic potential. *Nanomaterials.* (2024) 14:1854. 10.3390/nano14221854 39591094 PMC11597456

[54] Parveen S Gupta P Kumar S Banerjee M. Lipid polymer hybrid nanoparticles as potent vehicles for drug delivery in cancer therapeutics. *Med Drug Discov.* (2023) 20:100165. 10.1016/j.medidd.2023.100165

[55] Sadiq S Khan S Khan I Khan A Humayun M Wu P et al. A critical review on metal-organic frameworks (MOFs) based nanomaterials for biomedical applications: designing, recent trends, challenges, and prospects. *Heliyon.* (2024) 10:e25521. 10.1016/j.heliyon.2024.e25521 38356588 PMC10864983

[56] Abdelhamid M Khalifa H Ki M Pack S. Nanoengineered silica-based biomaterials for regenerative medicine. *Int J Mol Sci.* (2024) 25:6125. 10.3390/ijms25116125 38892312 PMC11172759

[57] Liu X He F Liu M. New opportunities of stimulus-responsive smart nanocarriers in cancer therapy. *Nano Mater Sci.* (2024): 10.1016/j.nanoms.2024.10.013

[58] Al Refaai K AlSawaftah N Abuwatfa W Husseini G. Drug release via ultrasound-activated nanocarriers for cancer treatment: a review. *Pharmaceutics.* (2024) 16:1383. 10.3390/pharmaceutics16111383 39598507 PMC11597164

[59] Karimi M Sahandi Zangabad P Ghasemi A Amiri M Bahrami M Malekzad H et al. Temperature-responsive smart nanocarriers for delivery of therapeutic agents: applications and recent advances. *ACS Appl Mater Interfaces.* (2016) 8:21107–33. 10.1021/acsami.6b00371 27349465 PMC5003094

[60] Mi P. Stimuli-responsive nanocarriers for drug delivery, tumor imaging, therapy and theranostics. *Theranostics.* (2020) 10:4557–88. 10.7150/thno.38069 32292515 PMC7150471

[61] Almadani I Almadani M AlSawaftah N Abuwatfa W Husseini G. Nanocarriers responsive to light—A review. *Micro.* (2024) 4:827–44. 10.3390/micro4040051

[62] Chu S Shi X Tian Y Gao F. pH-responsive polymer nanomaterials for tumor therapy. *Front Oncol.* (2022) 12:855019. 10.3389/fonc.2022.855019 35392227 PMC8980858

[63] Demina P Khaydukov K Babayeva G Varaksa P Atanova A Stepanov M et al. Upconversion nanoparticles intercalated in large polymer micelles for tumor imaging and chemo/photothermal therapy. *Int J Mol Sci.* (2023) 24:10574. 10.3390/ijms241310574 37445751 PMC10342109

[64] Zhang P Wu J Xiao F Zhao D Luan Y. Disulfide bond based polymeric drug carriers for cancer chemotherapy and relevant redox environments in mammals. *Med Res Rev.* (2018) 38:1485–510. 10.1002/med.21485 29341223

[65] Meng X Shen Y Zhao H Lu X Wang Z Zhao Y. Redox-manipulating nanocarriers for anticancer drug delivery: a systematic review. *J Nanobiotechnology.* (2024) 22:587. 10.1186/s12951-024-02859-w 39342211 PMC11438196

[66] Han S Li Z Zhu J Han K Zeng Z Hong W et al. Dual-pH sensitive charge-reversal polypeptide micelles for tumor-triggered targeting uptake and nuclear drug delivery. *Small.* (2015) 11:2543–54. 10.1002/smll.201402865 25626995

[67] Deng H Liu J Zhao X Zhang Y Liu J Xu S et al. PEG-b-PCL copolymer micelles with the ability of pH-controlled negative-to-positive charge reversal for intracellular delivery of doxorubicin. *Biomacromolecules.* (2014) 15:4281–92. 10.1021/bm501290t 25325531

[68] Li N Cai H Jiang L Hu J Bains A Hu J et al. Enzyme-sensitive and amphiphilic PEGylated dendrimer-paclitaxel prodrug-based nanoparticles for enhanced stability and anticancer efficacy. *ACS Appl Mater Interfaces.* (2017) 9:6865–77. 10.1021/acsami.6b15505 28112512

[69] Wang S Huang P Chen X. Stimuli-responsive programmed specific targeting in nanomedicine. *ACS Nano.* (2016) 10:2991–4. 10.1021/acsnano.6b00870 26881288 PMC5223089

[70] Xiao D Jia H Ma N Zhuo R Zhang XZ. A redox-responsive mesoporous silica nanoparticle capped with amphiphilic peptides by self-assembly for cancer targeting drug delivery. *Nanoscale.* (2015) 7:10071–7. 10.1039/c5nr02247a 25978679

[71] Chen Y Wang Y He L Wang Z Shen Y Cong H et al. Redox and pH double stimulus-responsive mesoporous silica nanoparticles for drug delivery. *Ferroelectrics.* (2019) 549:1–11. 10.1080/00150193.2019.1592538

[72] Palanikumar L Al-Hosani S Kalmouni M Nguyen V Ali L Pasricha R et al. pH-responsive high stability polymeric nanoparticles for targeted delivery of anticancer therapeutics. *Commun Biol.* (2020) 3:95. 10.1038/s42003-020-0817-4 32127636 PMC7054360

[73] Li Z Hu J Xu Q Chen S Jia H Sun Y et al. A redox-responsive drug delivery system based on RGD containing peptide-capped mesoporous silica nanoparticles. *J Mater Chem B.* (2015) 3:39–44. 10.1039/c4tb01533a 32261922

[74] Tian Y Lei M. Polydopamine-based composite nanoparticles with redox-labile polymer shells for controlled drug release and enhanced chemo-photothermal therapy. *Nanoscale Res Lett.* (2019) 14:186. 10.1186/s11671-019-3027-6 31147801 PMC6542907

[75] Yu J Chu X Hou Y. Stimuli-responsive cancer therapy based on nanoparticles. *Chem Commun.* (2014) 50:11614–30. 10.1039/c4cc03984j 25058003

[76] Qu H Yang L Yu J Dong T Rong M Zhang J et al. A redox responsive controlled release system using mesoporous silica nanoparticles capped with Au nanoparticles. *RSC Adv.* (2017) 7:35704–10. 10.1039/C7RA04444E

[77] Wang M Gong G Feng J Wang T Ding C Zhou B et al. Dual pH-mediated mechanized hollow zirconia nanospheres. *ACS Appl Mater Interfaces.* (2016) 8:23289–301. 10.1021/acsami.6b07603 27523904

[78] Hu Q Katti P Gu Z. Enzyme-responsive nanomaterials for controlled drug delivery. *Nanoscale.* (2014) 6:12273–86. 10.1039/c4nr04249b 25251024 PMC4425417

[79] Grünwald B Vandooren J Locatelli E Fiten P Opdenakker G Proost P et al. Matrix metalloproteinase-9 (MMP-9) as an activator of nanosystems for targeted drug delivery in pancreatic cancer. *J Control Release.* (2016) 239:39–48. 10.1016/j.jconrel.2016.08.016 27545397

[80] Kurul F Turkmen H Cetin A Topkaya S. Nanomedicine: how nanomaterials are transforming drug delivery, bio-imaging, and diagnosis. *Next Nanotechnol.* (2025) 7:100129. 10.1016/j.nxnano.2024.100129

[81] Guan X Xing S Liu Y. Engineered cell membrane-camouflaged nanomaterials for biomedical applications. *Nanomaterials.* (2024) 14:413. 10.3390/nano14050413 38470744 PMC10935217

[82] Liu H Su Y Jiang X Gao J. Cell membrane-coated nanoparticles: a novel multifunctional biomimetic drug delivery system. *Drug Deliv Transl Res.* (2023) 13:716–37. 10.1007/s13346-022-01252-0 36417162 PMC9684886

[83] Liu H Li Y Wang Y Zhang L Liang X Gao C et al. Red blood cells-derived components as biomimetic functional materials: matching versatile delivery strategies based on structure and function. *Bioact Mater.* (2025) 47:481–501. 10.1016/j.bioactmat.2025.01.021 40034412 PMC11872572

[84] Diez-Silva M Dao M Han J Lim C Suresh S. Shape and biomechanical characteristics of human red blood cells in health and disease. *MRS Bull.* (2010) 35:382–8. 10.1557/mrs2010.571 21151848 PMC2998922

[85] Berikkhanova K Taigulov E Bokebaev Z Kusainov A Tanysheva G Yedrissov A et al. Drug-loaded erythrocytes: modern approaches for advanced drug delivery for clinical use. *Heliyon.* (2024) 10:e23451. 10.1016/j.heliyon.2023.e23451 38192824 PMC10772586

[86] Zhang J Chen C Fu H Yu J Sun Y Huang H et al. MicroRNA-125a-loaded polymeric nanoparticles alleviate systemic lupus erythematosus by restoring effector/regulatory T cells balance. *ACS Nano.* (2020) 14:4414–29. 10.1021/acsnano.9b09998 32203665

[87] Guan F Wang R Yi Z Luo P Liu W Xie Y et al. Tissue macrophages: origin, heterogenity, biological functions, diseases and therapeutic targets. *Signal Transduct Target Ther.* (2025) 10:93. 10.1038/s41392-025-02124-y 40055311 PMC11889221

[88] Garanina A Vishnevskiy D Chernysheva A Valikhov M Malinovskaya J Lazareva P et al. Neutrophil as a carrier for cancer nanotherapeutics: a comparative study of liposome, PLGA, and magnetic nanoparticles delivery to tumors. *Pharmaceuticals.* (2023) 16:1564. 10.3390/ph16111564 38004431 PMC10674452

[89] Rosales C Demaurex N Lowell C Uribe-Querol E. Neutrophils: their role in innate and adaptive immunity. *J Immunol Res.* (2016) 2016:1469780. 10.1155/2016/1469780 27006954 PMC4783580

[90] Payamifar S Khalili Y Foroozandeh A Abdouss M Hasanzadeh M. Magnetic mesoporous silica nanoparticles as advanced polymeric scaffolds for efficient cancer chemotherapy: recent progress and challenges. *RSC Adv.* (2025) 15:16050–74. 10.1039/d5ra00948k 40370857 PMC12076205

[91] Petroni D Fabbri C Babboni S Menichetti L Basta G Del Turco S. Extracellular vesicles and intercellular communication: challenges for in vivo molecular imaging and tracking. *Pharmaceutics.* (2023) 15:1639. 10.3390/pharmaceutics15061639 37376087 PMC10301899

[92] Chavda V Pandya A Kumar L Raval N Vora L Pulakkat S et al. Exosome nanovesicles: a potential carrier for therapeutic delivery. *Nano Today.* (2023) 49:101771. 10.1016/j.nantod.2023.101771

[93] de Visser K Joyce J. The evolving tumor microenvironment: from cancer initiation to metastatic outgrowth. *Cancer Cell.* (2023) 41:374–403. 10.1016/j.ccell.2023.02.016 36917948

[94] Luo Y Chang L Lin P. Metal-based nanoparticles and the immune system: activation, inflammation, and potential applications. *Biomed Res Int.* (2015) 2015:143720. 10.1155/2015/143720 26125021 PMC4466342

[95] Pondman K Le Gac S Kishore U. Nanoparticle-induced immune response: health risk versus treatment opportunity? *Immunobiology.* (2023) 228:152317. 10.1016/j.imbio.2022.152317 36592542

[96] Qin W Huang G Chen Z Zhang Y. Nanomaterials in targeting cancer stem cells for cancer therapy. *Front Pharmacol.* (2017) 8:1. 10.3389/fphar.2017.00001 28149278 PMC5241315

[97] Ciepła J Smolarczyk R. Tumor hypoxia unveiled: insights into microenvironment, detection tools and emerging therapies. *Clin Exp Med.* (2024) 24:235. 10.1007/s10238-024-01501-1 39361163 PMC11449960

[98] George Joy J Sharma G Kim J. Tailoring polymeric nanocarriers for hypoxia-specific drug release: insights into design and applications in clinics. *Chem Eng J.* (2024) 496:153978. 10.1016/j.cej.2024.153978

[99] Bogdanov A Bogdanov A Chubenko V Volkov N Moiseenko F Moiseyenko V. Tumor acidity: from hallmark of cancer to target of treatment. *Front Oncol.* (2022) 12:979154. 10.3389/fonc.2022.979154 36106097 PMC9467452

[100] Karimi M Eslami M Sahandi-Zangabad P Mirab F Farajisafiloo N Shafaei Z et al. pH-Sensitive stimulus-responsive nanocarriers for targeted delivery of therapeutic agents. *Wiley Interdiscip Rev Nanomed Nanobiotechnol.* (2016) 8:696–716. 10.1002/wnan.1389 26762467 PMC4945487

[101] Henke E Nandigama R Ergün S. Extracellular matrix in the tumor microenvironment and its impact on cancer therapy. *Front Mol Biosci.* (2019) 6:160. 10.3389/fmolb.2019.00160 32118030 PMC7025524

[102] Emran T Shahriar A Mahmud A Rahman T Abir M Siddiquee M et al. Multidrug resistance in cancer: understanding molecular mechanisms, immunoprevention and therapeutic approaches. *Front Oncol.* (2022) 12:891652. 10.3389/fonc.2022.891652 35814435 PMC9262248

[103] Gao L Meng F Yang Z Lafuente-Merchan M Fernández L Cao Y et al. Nano-drug delivery system for the treatment of multidrug-resistant breast cancer: current status and future perspectives. *Biomed Pharmacother.* (2024) 179:117327. 10.1016/j.biopha.2024.117327 39216449

[104] Attia M Kijanka G Nguyen N Zhang J An H. Advances and prospects of RNA delivery nanoplatforms for cancer therapy. *Acta Pharm Sin B.* (2025) 15:52–96. 10.1016/j.apsb.2024.09.009 40041887 PMC11873661

[105] Taibi T Cheon S Perna F Vu L. mRNA-based therapeutic strategies for cancer treatment. *Mol Ther.* (2024) 32:2819–34. 10.1016/j.ymthe.2024.04.035 38702886 PMC11403232

[106] Hao Y Ji Z Zhou H Wu D Gu Z Wang D et al. Lipid-based nanoparticles as drug delivery systems for cancer immunotherapy. *MedComm.* (2023) 4:e339. 10.1002/mco2.339 37560754 PMC10407046

[107] Byrnes A Dominguez S Yen C Laufer B Foreman O Reichelt M et al. Lipid nanoparticle delivery limits antisense oligonucleotide activity and cellular distribution in the brain after intracerebroventricular injection. *Mol Ther Nucleic Acids.* (2023) 32:773–93. 10.1016/j.omtn.2023.05.005 37346977 PMC10280097

[108] Sharma A Lee Y Bat-Ulzii A Bhattacharya M Chakraborty C Lee S. Recent advances of metal-based nanoparticles in nucleic acid delivery for therapeutic applications. *J Nanobiotechnology.* (2022) 20:501. 10.1186/s12951-022-01650-z 36434667 PMC9700905

[109] Sinani G Durgun M Cevher E Özsoy Y. Polymeric-micelle-based delivery systems for nucleic acids. *Pharmaceutics.* (2023) 15:2021. 10.3390/pharmaceutics15082021 37631235 PMC10457940

[110] Mollé L Smyth C Yuen D Johnston A. Nanoparticles for vaccine and gene therapy: overcoming the barriers to nucleic acid delivery. *Wiley Interdiscip Rev Nanomed Nanobiotechnol.* (2022) 14:e1809. 10.1002/wnan.1809 36416028 PMC9786906

[111] Alsaab H Alharbi F Alhibs A Alanazi N Alshehri B Saleh M et al. PLGA-based nanomedicine: history of advancement and development in clinical applications of multiple diseases. *Pharmaceutics.* (2022) 14:2728. 10.3390/pharmaceutics14122728 36559223 PMC9786338

[112] Wu Z Zhang H Yan J Wei Y Su J. Engineered biomembrane-derived nanoparticles for nanoscale theranostics. *Theranostics.* (2023) 13:20–39. 10.7150/thno.76894 36593970 PMC9800735

[113] Barkat A Beg S Panda S Alharbi K Rahman M Ahmed FJ. Functionalized mesoporous silica nanoparticles in anticancer therapeutics. *Semin Cancer Biol.* (2021) 69:365–75. 10.1016/j.semcancer.2019.08.022 31442571

[114] Overchuk M Weersink R Wilson B Zheng G. Photodynamic and photothermal therapies: synergy opportunities for nanomedicine. *ACS Nano.* (2023) 17:7979–8003. 10.1021/acsnano.3c00891 37129253 PMC10173698

[115] Zhao L Liu Y Chang R Xing R Yan X. Supramolecular photothermal nanomaterials as an emerging paradigm toward precision cancer therapy. *Adv Funct Mater.* (2019) 29:1806877. 10.1002/adfm.201806877

[116] Li J Wang S Fontana F Tapeinos C Shahbazi M Han H et al. Nanoparticles-based phototherapy systems for cancer treatment: current status and clinical potential. *Bioact Mater.* (2023) 23:471–507. 10.1016/j.bioactmat.2022.11.013 36514388 PMC9727595

[117] Shabnum S Siranjeevi R Raj C Saravanan A Vickram A Chopra H et al. Advancements in nanotechnology-driven photodynamic and photothermal therapies: mechanistic insights and synergistic approaches for cancer treatment. *RSC Adv.* (2024) 14:38952–95. 10.1039/d4ra07114j 39659608 PMC11629304

[118] Badir A Refki S Sekkat Z. Utilizing gold nanoparticles in plasmonic photothermal therapy for cancer treatment. *Heliyon.* (2025) 11:e42738. 10.1016/j.heliyon.2025.e42738 40084020 PMC11904586

[119] Basak S Das T. Liposome-based drug delivery systems: from laboratory research to industrial production—instruments and challenges. *ChemEngineering.* (2025) 9:56. 10.3390/chemengineering9030056

[120] Giustini A Petryk A Cassim S Tate J Baker I Hoopes P. Magnetic nanoparticle hyperthermia in cancer treatment. *Nano Life.* (2010) 1: 10.1142/S1793984410000067 24348868 PMC3859910

[121] Rastogi V Yadav P Bhattacharya S Mishra A Verma N Verma A et al. Carbon nanotubes: an emerging drug carrier for targeting cancer cells. *J Drug Deliv.* (2014) 2014:670815. 10.1155/2014/670815 24872894 PMC4020363

[122] Mimona M Rimon M Zohura F Sony J Rim S Arup M et al. Quantum dot nanomaterials: empowering advances in optoelectronic devices. *Chem Eng J Adv.* (2025) 21:100704. 10.1016/j.ceja.2025.100704

[123] Pathak R Bhatt S Punetha V Punetha M. Chitosan nanoparticles and based composites as a biocompatible vehicle for drug delivery: a review. *Int J Biol Macromol.* (2023) 253:127369. 10.1016/j.ijbiomac.2023.127369 37839608

[124] Sokolova V Epple M. Biological and medical applications of calcium phosphate nanoparticles. *Chemistry.* (2021) 27:7471–88. 10.1002/chem.202005257 33577710 PMC8251768

[125] Cucinotto I Fiorillo L Gualtieri S Arbitrio M Ciliberto D Staropoli N et al. Nanoparticle albumin bound Paclitaxel in the treatment of human cancer: nanodelivery reaches prime-time? *J Drug Deliv.* (2013) 2013:905091. 10.1155/2013/905091 23738077 PMC3659516

[126] Hossen S Hossain M Basher M Mia M Rahman M Uddin M. Smart nanocarrier-based drug delivery systems for cancer therapy and toxicity studies: a review. *J Adv Res.* (2019) 15:1–18. 10.1016/j.jare.2018.06.005 30581608 PMC6300464

[127] Garg S Garg G Bisht A Dubey A Sharma L. Chapter 14 - Nanotoxicity and challenges in stimuli-responsive vesicular carriers targeting tumor. In: Jain A Mody N Palakurthi S editors. *Tumor-Targeting with Stimuli-Responsive Vesicular Nanocarriers.* Cambridge, MA: Academic Press (2025). p. 373–408.

[128] Yasir M Mishra R Tripathi A Maurya R Shahi A Zaki M et al. Theranostics: a multifaceted approach utilizing nano-biomaterials. *Discov Nano.* (2024) 19:35. 10.1186/s11671-024-03979-w 38407670 PMC10897124

[129] Zhou T Cheng Q Zhang L Zhang D Li L Jiang T et al. Ferrocene-functionalized core–shell lanthanide-doped upconversion nanoparticles: NIR light promoted chemodynamic therapy and luminescence imaging of solid tumors. *Chem Eng J.* (2022) 438:135637. 10.1016/j.cej.2022.135637

[130] Khan I Saeed K Khan I. Nanoparticles: properties, applications and toxicities. *Arab J Chem.* (2019) 12:908–31. 10.1016/j.arabjc.2017.05.011

[131] Raheem M Rahim M Gul I Zhong X Xiao C Zhang H et al. Advances in nanoparticles-based approaches in cancer theranostics. *OpenNano.* (2023) 12:100152. 10.1016/j.onano.2023.100152

[132] Ferreira C Goel S Ehlerding E Rosenkrans Z Jiang D Sun T et al. Ultrasmall porous silica nanoparticles with enhanced pharmacokinetics for cancer theranostics. *Nano Lett.* (2021) 21:4692–9. 10.1021/acs.nanolett.1c00895 34029471 PMC8265214

[133] Wang L Li D Hao Y Niu M Hu Y Zhao H et al. Gold nanorod-based poly(lactic-co-glycolic acid) with manganese dioxide core-shell structured multifunctional nanoplatform for cancer theranostic applications. *Int J Nanomedicine.* (2017) 12:3059–75. 10.2147/IJN.S128844 28450782 PMC5399988

[134] Li Y Jiang C Zhang D Wang Y Ren X Ai K et al. Targeted polydopamine nanoparticles enable photoacoustic imaging guided chemo-photothermal synergistic therapy of tumor. *Acta Biomater.* (2017) 47:124–34. 10.1016/j.actbio.2016.10.010 27721008

[135] Gholami L Tafaghodi M Abbasi B Daroudi M Kazemi Oskuee R. Preparation of superparamagnetic iron oxide/doxorubicin loaded chitosan nanoparticles as a promising glioblastoma theranostic tool. *J Cell Physiol.* (2019) 234:1547–59. 10.1002/jcp.27019 30145790

[136] Feng L Cheng L Dong Z Tao D Barnhart T Cai W et al. Theranostic liposomes with hypoxia-activated prodrug to effectively destruct hypoxic tumors post-photodynamic therapy. *ACS Nano.* (2017) 11:927–37. 10.1021/acsnano.6b07525 28027442 PMC5372701

[137] Zhao W Li T Long Y Guo R Sheng Q Lu Z et al. Self-promoted albumin-based nanoparticles for combination therapy against metastatic breast cancer via a hyperthermia-induced platelet bridge. *ACS Appl Mater Interfaces.* (2021) 13:25701–14. 10.1021/acsami.1c04442 34041901

[138] Xiao W Luo J Jain T Riggs J Tseng H Henderson P et al. Biodistribution and pharmacokinetics of a telodendrimer micellar paclitaxel nanoformulation in a mouse xenograft model of ovarian cancer. *Int J Nanomedicine.* (2012) 7:1587–97. 10.2147/IJN.S29306 22605931 PMC3352867

[139] De Silva L Fu J Htar T Wan Kamal W Kasbollah A Muniyandy S et al. Biodistribution study of niosomes in tumor-implanted BALB/C mice using scintigraphic imaging. *Front Pharmacol.* (2021) 12:778396. 10.3389/fphar.2021.778396 35069200 PMC8777053

[140] Dejeu I Vicaş LG Marian E Ganea M Frenţ OD Maghiar PB et al. Innovative approaches to enhancing the biomedical properties of liposomes. *Pharmaceutics.* (2024) 16:1525. 10.3390/pharmaceutics16121525 39771504 PMC11728823

[141] Bui T Mei H Sang R Ortega D Deng W. Advancements and challenges in developing in vivo CAR T cell therapies for cancer treatment. *EBioMedicine.* (2024) 106:105266. 10.1016/j.ebiom.2024.105266 39094262 PMC11345408

[142] McCarthy E. The toxins of William B. Coley and the treatment of bone and soft-tissue sarcomas. *Iowa Orthop J.* (2006) 26:154–8. 16789469 PMC1888599

[143] Gamboa J Leong K. In vitro and in vivo models for the study of oral delivery of nanoparticles. *Adv Drug Deliv Rev.* (2013) 65:800–10. 10.1016/j.addr.2013.01.003 23415952 PMC3773489

[144] Pulit-Prociak J Długosz O Staroń A Domagała D Pociecha K Grabowski M et al. In vitro and in vivo studies of titanium dioxide nanoparticles with galactose coating as a prospective drug carrier. *ACS Omega.* (2024) 9:36220–31. 10.1021/acsomega.4c02232 39220526 PMC11360011

[145] Sun M Lee J Chen Y Hoshino K. Studies of nanoparticle delivery with in vitro bio-engineered microtissues. *Bioact Mater.* (2020) 5:924–37. 10.1016/j.bioactmat.2020.06.016 32637755 PMC7330434

[146] Rima M Dakramanji M El Hayek E El Khoury T Fajloun Z Rima M. Unveiling the wonders of bacteria-derived extracellular vesicles: from fundamental functions to beneficial applications. *Heliyon.* (2025) 11:e42509. 10.1016/j.heliyon.2025.e42509 40028522 PMC11869109

[147] Lao J Madani J Puértolas T Alvarez M Hernández A Pazo-Cid R et al. Liposomal Doxorubicin in the treatment of breast cancer patients: a review. *J Drug Deliv.* (2013) 2013:456409. 10.1155/2013/456409 23634302 PMC3619536

[148] Kneidl B Peller M Winter G Lindner L Hossann M. Thermosensitive liposomal drug delivery systems: state of the art review. *Int J Nanomedicine.* (2014) 9:4387–98. 10.2147/IJN.S49297 25258529 PMC4172103

[149] Kundranda M Niu J. Albumin-bound paclitaxel in solid tumors: clinical development and future directions. *Drug Des Devel Ther.* (2015) 9:3767–77. 10.2147/DDDT.S88023 26244011 PMC4521678

[150] Estapé Senti M García Del Valle L Schiffelers RM. mRNA delivery systems for cancer immunotherapy: lipid nanoparticles and beyond. *Adv Drug Deliv Rev.* (2024) 206:115190. 10.1016/j.addr.2024.115190 38307296

[151] Pathak P Hess R Weiss M. Liposomal vincristine for relapsed or refractory Ph-negative acute lymphoblastic leukemia: a review of literature. *Ther Adv Hematol.* (2014) 5:18–24. 10.1177/2040620713519016 24490021 PMC3891290

[152] Nel J Elkhoury K Velot E Bianchi A Acherar S Francius G et al. Functionalized liposomes for targeted breast cancer drug delivery. *Bioact Mater.* (2023) 24:401–37. 10.1016/j.bioactmat.2022.12.027 36632508 PMC9812688

[153] Money-Kyrle J Bates F Ready J Gazzard B Phillips R Boag F. Liposomal daunorubicin in advanced Kaposi’s sarcoma: a phase II study. *Clin Oncol.* (1993) 5:367–71. 10.1016/s0936-6555(05)80088-3 8305357

[154] Sachdeva V Monga A Vashisht R Singh D Singh A Bedi N. Iron oxide nanoparticles: the precise strategy for targeted delivery of genes, oligonucleotides and peptides in cancer therapy. *J Drug Deliv.* (2022) 74:103585. 10.1016/j.jddst.2022.103585

[155] Kim D Kim S Kim H Kim S Shin S Kim J et al. Multicenter phase II trial of Genexol-PM, a novel Cremophor-free, polymeric micelle formulation of paclitaxel, with cisplatin in patients with advanced non-small-cell lung cancer. *Ann Oncol.* (2007) 18:2009–14. 10.1093/annonc/mdm374 17785767

[156] Ma P Wang G Men K Li C Gao N Li L. Advances in clinical application of nanoparticle-based therapy for cancer treatment: a systematic review. *Nano TransMed.* (2024) 3:100036. 10.1016/j.ntm.2024.100036

[157] Liu Q Zou J Chen Z He W Wu W. Current research trends of nanomedicines. *Acta Pharm Sin B.* (2023) 13:4391–416. 10.1016/j.apsb.2023.05.018 37969727 PMC10638504

[158] Namiot ED Sokolov A Chubarev V Tarasov V Schiöth H. Nanoparticles in clinical trials: analysis of clinical trials, FDA approvals and use for COVID-19 vaccines. *Int J Mol Sci.* (2023) 24:787. 10.3390/ijms24010787 36614230 PMC9821409

[159] Ajith S Almomani F Elhissi A Husseini G. Nanoparticle-based materials in anticancer drug delivery: current and future prospects. *Heliyon.* (2023) 9:e21227. 10.1016/j.heliyon.2023.e21227 37954330 PMC10637937

[160] Amreddy N Babu A Muralidharan R Panneerselvam J Srivastava A Ahmed R et al. Recent advances in nanoparticle-based cancer drug and gene delivery. *Adv Cancer Res.* (2018) 137:115–70. 10.1016/bs.acr.2017.11.003 29405974 PMC6550462

[161] Soetaert F Korangath P Serantes D Fiering S Ivkov R. Cancer therapy with iron oxide nanoparticles: agents of thermal and immune therapies. *Adv Drug Deliv Rev.* (2020) 163-164:65–83. 10.1016/j.addr.2020.06.025 32603814 PMC7736167

[162] Droste M Thakur B Eliceiri B. Tumor-derived extracellular vesicles and the immune system-lessons from immune-competent mouse-tumor models. *Front Immunol.* (2020) 11:606859. 10.3389/fimmu.2020.606859 33391275 PMC7772428

[163] Chung Y Cai H Steinmetz N. Viral nanoparticles for drug delivery, imaging, immunotherapy, and theranostic applications. *Adv Drug Deliv Rev.* (2020) 156:214–35. 10.1016/j.addr.2020.06.024 32603813 PMC7320870

[164] Wu J Ko S Lee E Son E Kang G Hur S et al. Gold nanoparticles in imaging: advances, applications, and future perspectives. *Appl Spectrosc Rev.* (2025) 60:978–1017. 10.1080/05704928.2025.2495022

[165] Zhu J Lee H Huang R Zhou J Zhang J Yang X et al. Harnessing nanotechnology for cancer treatment. *Front Bioeng Biotechnol.* (2025) 12:1514890. 10.3389/fbioe.2024.1514890 39902172 PMC11788409

[166] Mundekkad D Cho W. Nanoparticles in clinical translation for cancer therapy. *Int J Mol Sci.* (2022) 23:1685. 10.3390/ijms23031685 35163607 PMC8835852

[167] Farjadian F Ghasemi A Gohari O Roointan A Karimi M Hamblin M. Nanopharmaceuticals and nanomedicines currently on the market: challenges and opportunities. *Nanomedicine.* (2019) 14:93–126. 10.2217/nnm-2018-0120 30451076 PMC6391637

[168] Cheow W Hadinoto K. Factors affecting drug encapsulation and stability of lipid-polymer hybrid nanoparticles. *Colloids Surf B Biointerfaces.* (2011) 85:214–20. 10.1016/j.colsurfb.2011.02.033 21439797

[169] Hafeez M Celia C Petrikaite V. Challenges towards targeted drug delivery in cancer nanomedicines. *Processes.* (2021) 9:1527. 10.3390/pr9091527

[170] Gavas S Quazi S Karpiński T. Nanoparticles for cancer therapy: current progress and challenges. *Nanoscale Res Lett.* (2021) 16:173. 10.1186/s11671-021-03628-6 34866166 PMC8645667

[171] Rodriguez P Harada T Christian D Pantano D Tsai R Discher D. Minimal Self peptides that inhibit phagocytic clearance and enhance delivery of nanoparticles. *Science.* (2013) 339:971–5. 10.1126/science.1229568 23430657 PMC3966479

[172] Sanna V Pala N Sechi M. Targeted therapy using nanotechnology: focus on cancer. *Int J Nanomedicine.* (2014) 9:467–83. 10.2147/IJN.S36654 24531078 PMC3896284

[173] Salvioni L Rizzuto M Bertolini J Pandolfi L Colombo M Prosperi D. Thirty years of cancer nanomedicine: success, frustration, and hope. *Cancers.* (2019) 11:1855. 10.3390/cancers11121855 31769416 PMC6966668

[174] Duan X Li Y. Physicochemical characteristics of nanoparticles affect circulation, biodistribution, cellular internalization, and trafficking. *Small.* (2013) 9:1521–32. 10.1002/smll.201201390 23019091

[175] Mosquera J García I Liz-Marzán L. Cellular uptake of nanoparticles versus small molecules: a matter of size. *Acc Chem Res.* (2018) 51:2305–13. 10.1021/acs.accounts.8b00292 30156826

[176] Agrahari V Agrahari V. Facilitating the translation of nanomedicines to a clinical product: challenges and opportunities. *Drug Discov Today.* (2018) 23:974–91. 10.1016/j.drudis.2018.01.047 29406263

[177] Wang S Cheng K Chen K Xu C Ma P Dang G et al. Nanoparticle-based medicines in clinical cancer therapy. *Nano Today.* (2022) 45:101512. 10.1016/j.nantod.2022.101512

[178] Rasool M Malik A Waquar S Arooj M Zahid S Asif M et al. New challenges in the use of nanomedicine in cancer therapy. *Bioengineered.* (2022) 13:759–73. 10.1080/21655979.2021.2012907 34856849 PMC8805951

[179] Zhou Y Zhang X Zhang J Cheng Y Wu J Yu J et al. Molecular weight characterization of cellulose using ionic liquids. *Polymer Testing.* (2021) 93:106985. 10.1016/j.polymertesting.2020.106985

[180] Kus-Liśkiewicz M Fickers P Ben Tahar I. Biocompatibility and cytotoxicity of gold nanoparticles: recent advances in methodologies and regulations. *Int J Mol Sci.* (2021) 22:10952. 10.3390/ijms222010952 34681612 PMC8536023

[181] Singh G Thakur N Kumar R. Nanoparticles in drinking water: assessing health risks and regulatory challenges. *Sci Total Environ.* (2024) 949:174940. 10.1016/j.scitotenv.2024.174940 39047836

[182] Ebbesen M Jensen T. Nanomedicine: techniques, potentials, and ethical implications. *J Biomed Biotechnol.* (2006) 2006:51516. 10.1155/JBB/2006/51516 17489016 PMC1779503

[183] Sun L Liu H Ye Y Lei Y Islam R Tan S et al. Smart nanoparticles for cancer therapy. *Signal Transduct Target Ther.* (2023) 8:418. 10.1038/s41392-023-01642-x 37919282 PMC10622502

[184] Spencer D Puranik A Peppas N. Intelligent nanoparticles for advanced drug delivery in cancer treatment. *Curr Opin Chem Eng.* (2015) 7:84–92. 10.1016/j.coche.2014.12.003 25621200 PMC4303181

[185] Elemike E Onunkwo I Ughumiakpor O Alawuru F Mukoro A Ishom P et al. Bio-nanomaterials: promising anticancer properties and treatment strategies. *Nano TransMed.* (2025) 4:100076. 10.1016/j.ntm.2025.100076

[186] Biswas R Alam M Sarkar A Haque M Hasan M Hoque M. Application of nanotechnology in food: processing, preservation, packaging and safety assessment. *Heliyon.* (2022) 8:e11795. 10.1016/j.heliyon.2022.e11795 36444247 PMC9699984

[187] Jagtiani E. Advancements in nanotechnology for food science and industry. *Food Frontiers.* (2022) 3:56–82. 10.1002/fft2.104

[188] McClements D. Delivery by design (DbD): a standardized approach to the development of efficacious nanoparticle- and microparticle-based delivery systems. *Compr Rev Food Sci Food Saf.* (2018) 17:200–19. 10.1111/1541-4337.12313 33350064

[189] Faridi Esfanjani A Jafari S. Biopolymer nano-particles and natural nano-carriers for nano-encapsulation of phenolic compounds. *Colloids Surf B Biointerfaces.* (2016) 146:532–43. 10.1016/j.colsurfb.2016.06.053 27419648

[190] Pateiro M Gómez B Munekata P Barba F Putnik P Kovačević D et al. Nanoencapsulation of promising bioactive compounds to improve their absorption, stability, functionality and the appearance of the final food products. *Molecules.* (2021) 26:1547. 10.3390/molecules26061547 33799855 PMC7999092

[191] Paidari S Esmaeili Y Ibrahim S Vahedi S Al-Hilifi S Zamindar N. *Application of Nanoparticles to Enhance the Microbial Quality and Shelf Life of Food Products. Microbial Biotechnology in the Food Industry: Advances, Challenges, and Potential Solutions.* Berlin: Springer (2024). p. 75–102. 10.1007/978-3-031-41729-0_5

[192] da Costa N Libardi N Schambeck C Filho P da Costa R. Impact of additive application on the establishment of fast and stable aerobic granulation. *Appl Microbiol Biotechnol.* (2020) 104:5697–709. 10.1007/s00253-020-10657-1 32415318

[193] Rhim J Park H Ha C. Bio-nanocomposites for food packaging applications. *Prog Polym Sci.* (2013) 38:1629–52. 10.1016/j.progpolymsci.2013.05.008

[194] Taherimehr M YousefniaPasha H Tabatabaeekoloor R Pesaranhajiabbas E. Trends and challenges of biopolymer-based nanocomposites in food packaging. *Compr Rev Food Sci Food Saf.* (2021) 20:5321–44. 10.1111/1541-4337.12832 34611989

[195] Kuèuk N Primožič M Knez Ž Leitgeb M. Sustainable biodegradable biopolymer-based nanoparticles for healthcare applications. *Int J Mol Sci.* (2023) 24:3188. 10.3390/ijms24043188 36834596 PMC9964453

[196] Hussain S Akhter R Maktedar S. Advancements in sustainable food packaging: from eco-friendly materials to innovative technologies. *Sustain Food Technol.* (2024) 2:1297–364. 10.1039/d4fb00084f

